# Efficacy and Safety of Respiratory Syncytial Virus (RSV) Prefusion F Protein Vaccine (RSVPreF3 OA) in Older Adults Over 2 RSV Seasons

**DOI:** 10.1093/cid/ciae010

**Published:** 2024-01-22

**Authors:** Michael G Ison, Alberto Papi, Eugene Athan, Robert G Feldman, Joanne M Langley, Dong-Gun Lee, Isabel Leroux-Roels, Federico Martinon-Torres, Tino F Schwarz, Richard N van Zyl-Smit, Céline Verheust, Nancy Dezutter, Olivier Gruselle, Laurence Fissette, Marie-Pierre David, Lusine Kostanyan, Veronica Hulstrøm, Aurélie Olivier, Marie Van der Wielen, Dominique Descamps, for the AReSVi-006 Study Group, Mark Adams, Michael Adams, Clara Agutu, Elaine Jacqueline Akite, Ingrid Alt, Charles Andrews, Rafaelle Antonelli-Incalzi, Asmik Asatryan, Ghazaleh Bahrami, Elena Bargagli, Qasim Bhorat, Paul Bird, Przemyslaw Borowy, Celine Boutry, Carles Brotons Cuixart, David Browder, Judith Brown, Erik Buntinx, Donald Cameron, Laura Campora, Cyrille Cartier, Kenneth Chinsky, Melissa Choi, Eun-Ju Choo, Delphine Collete, Maria Corral Carrillo, Susanna Cuadripani, Matthew G Davis, Magali de Heusch, Ferdinandus de Looze, Marc De Meulemeester, Ferdinando De Negri, Nathalie de Schrevel, David DeAtkine, Viktoriya Dedkova, Peter Dzongowski, Tamara Eckermann, Brandon Essink, Karen Faulkner, Murdo Ferguson, Gregory Fuller, Isabel Maria Galan Melendez, Ivan Gentile, Wayne Ghesquiere, Doria Grimard, Scott Halperin, Amardeep Heer, Laura Helman, Andre Hotermans, Tomas Jelinek, Jackie Kamerbeek, Hyo Youl Kim, Murray Kimmel, Mark Koch, Satu Kokko, Susanna Koski, Shady Kotb, Antonio Lalueza, Jin-Soo Lee, Muriel Lins, Johannes Lombaard, Akbar Mahomed, Mario Malerba, Celine Marechal, Sandie Marion, Jean-Benoit Martinot, Cristina Masuet-Aumatell, Damien McNally, Carlos Eduardo Medina Pech, Jorge Mendez Galvan, Lise Mercati, Narcisa Elena Mesaros, Dieter Mesotten, Essack Mitha, Kathryn Mngadi, Beate Moeckesch, Barnaby Montgomery, Linda Murray, Rhiannon Nally, Silvia Narejos Perez, Joseph Newberg, Paul Nugent, Dolores Ochoa Mazarro, Harunori Oda, Maurizio Orso, Jacinto Ortiz Molina, Tatiana Pak, Dae Won Park, Meenakshi Patel, Minesh Patel, Anna Maria Pedro Pijoan, Alberto Borobia Perez, Lina Perez-Breva, Merce Perez Vera, Claudia Pileggi, Fabrizio Pregliasco, Carol Pretswell, Dean Quinn, Michele Reynolds, Viktor Romanenko, Jeffrey Rosen, Nathalie Roy, Belen Ruiz Antoran, Vardine Sahakyan, Hideaki Sakata, Joachim Sauter, Axel Schaefer, Izabela Sein Anand, Jose Antonio Serra Rexach, David Shu, Andres Siig, William Simon, Svetlana Smakotina, Katie Steenackers, Brigitte Stephan, Silvio Tafuri, Kenji Takazawa, Guy Tellier, Wim Terryn, Leslie Tharenos, Nick Thomas, Nicole Toursarkissian, Benita Ukkonen, Noah Vale, Pieter-Jan Van Landegem, Carline Vanden Abeele, Lode Vermeersch, Francesco Vitale, Olga Voloshyna, Judith White, Seong-Heon Wie, Jonathan Wilson, Pedro Ylisastigui, Manuel Zocco

**Affiliations:** Bethesda, Maryland, USA; Pulmonary Division, University of Ferrara, St. Anna University Hospital, Ferrara, Italy; Barwon Health, University Hospital Geelong, Geelong, Australia; Centre for Innovation in Infectious Diseases and Immunology Research, Deakin University, Geelong, Australia; Senior Clinical Trials, Inc, Laguna Hills, California, USA; Canadian Center for Vaccinology, Dalhousie University, IWK Health and Nova Scotia Health, Halifax, Canada; Division of Infectious Diseases, Department of Internal Medicine, Vaccine Bio Research Institute, College of Medicine, The Catholic University of Korea, Seoul, South Korea; Center for Vaccinology, Ghent University and Ghent University Hospital, Ghent, Belgium; Translational Pediatrics and Infectious Diseases, Pediatrics Department, Hospital Clínico Universitario de Santiago, Santiago de Compostela, Spain; Genetics, Vaccines, Infectious Diseases, and Pediatrics Research Group, Instituto de Investigación Sanitaria de Santiago, Universidad de Santiago de Compostela, Santiago de Compostela, Spain; Consorcio Centro de Investigación Biomédica en Red de Enfermedades Respiratorias, Instituto de Salud Carlos III, Madrid, Spain; Institute of Laboratory Medicine and Vaccination Center, Klinikum Würzburg Mitte, Campus Juliusspital, Würzburg, Germany; Division of Pulmonology and University of Cape Town Lung Institute, Department of Medicine, University of Cape Town and Groote Schuur Hospital, Cape Town, South Africa; GSK, Wavre, Belgium; GSK, Wavre, Belgium; GSK, Wavre, Belgium; GSK, Wavre, Belgium; GSK, Wavre, Belgium; GSK, Wavre, Belgium; GSK, Wavre, Belgium; GSK, Wavre, Belgium; GSK, Wavre, Belgium; GSK, Wavre, Belgium

**Keywords:** respiratory syncytial virus prefusion F protein vaccine, older adults, RSV

## Abstract

**Background:**

The adjuvanted RSV prefusion F protein–based vaccine (RSVPreF3 OA) was efficacious against RSV-related lower respiratory tract disease (RSV-LRTD) in ≥60-years-olds over 1 RSV season. We evaluated efficacy and safety of 1 RSVPreF3 OA dose and of 2 RSVPreF3 OA doses given 1 year apart against RSV-LRTD over 2 RSV seasons post–dose 1.

**Methods:**

In this phase 3, blinded trial, ≥60-year-olds were randomized (1:1) to receive RSVPreF3 OA or placebo pre–season 1. RSVPreF3 OA recipients were re-randomized (1:1) to receive a second RSVPreF3 OA dose (RSV_revaccination group) or placebo (RSV_1dose group) pre–season 2; participants who received placebo pre–season 1 received placebo pre–season 2 (placebo group). Efficacy of both vaccine regimens against RSV-LRTD was evaluated over 2 seasons combined (confirmatory secondary objective, success criterion: lower limits of 2-sided CIs around efficacy estimates >20%).

**Results:**

The efficacy analysis comprised 24 967 participants (RSV_1dose: 6227; RSV_revaccination: 6242; placebo: 12 498). Median efficacy follow-up was 17.8 months. Efficacy over 2 seasons of 1 RSVPreF3 OA dose was 67.2% (97.5% CI: 48.2–80.0%) against RSV-LRTD and 78.8% (95% CI: 52.6–92.0%) against severe RSV-LRTD. Efficacy over 2 seasons of a first dose followed by revaccination was 67.1% (97.5% CI: 48.1–80.0%) against RSV-LRTD and 78.8% (95% CI: 52.5–92.0%) against severe RSV-LRTD. Reactogenicity/safety of the revaccination dose were similar to dose 1.

**Conclusions:**

One RSVPreF3 OA dose was efficacious against RSV-LRTD over 2 RSV seasons in ≥60-year-olds. Revaccination 1 year post–dose 1 was well tolerated but did not seem to provide additional efficacy benefit in the overall study population.

**Clinical Trials Registration:**

ClinicalTrials.gov: NCT04886596.

## Teaser

One RSVPreF3 OA dose was efficacious against respiratory syncytial virus (RSV)–related lower respiratory tract disease during at least 2 RSV seasons in ≥60-year-olds. RSVPreF3 OA revaccination 1 year post–dose 1 did not seem to provide additional efficacy benefit. Optimal timing of revaccination remains to be determined.

Respiratory syncytial virus (RSV) is a contagious pathogen that causes seasonal epidemics of acute respiratory illness (ARI) [1]. RSV infection does not confer robust long-term immunity, and reinfections are common throughout life [2, 3]. While infection typically causes mild symptoms in adults, older adults and those with certain chronic conditions (like cardiorespiratory diseases or diabetes) are at increased risk of severe lower respiratory tract disease (LRTD), hospitalization, cardiorespiratory complications, and death due to RSV [4–9].

Two vaccines—both based on RSV fusion protein (F) stabilized in its prefusion conformation—have recently been authorized to prevent RSV-LRTD in individuals aged 60 years and older [10–13]. The ongoing AReSVi-006 trial, which studies 1 of these vaccines (RSVPreF3 OA; Arexvy; GSK), showed that RSVPreF3 OA had an acceptable safety profile and high efficacy against RSV-LRTD (82.6%), severe RSV-LRTD (94.1%), and RSV-ARI (71.7%) over 1 RSV season [14]. Efficacy was observed in different age groups and in participants with co-existing medical conditions [14, 15].

The AReSVi-006 trial also investigates RSVPreF3 OA efficacy and safety over multiple RSV seasons. We report the efficacy and safety over 2 seasons of 1 RSVPreF3 OA dose and of a first dose followed by revaccination 1 year later.

## METHODS

### Trial Design and Participants

The AReSVi-006 trial is a phase 3, randomized, placebo-controlled trial (NCT04886596) that is ongoing in 17 countries. Adults aged 60 years or older were enrolled (after providing informed consent) between 25 May 2021 and 31 January 2022. Individuals with chronic medical conditions (with/without specific treatment) were eligible if the investigator considered their conditions medically stable. Detailed eligibility criteria were reported previously [14].

Results based on data available until April 2022 (end of the first Northern Hemisphere RSV season), including the primary outcome, have been published [14]. Results disclosed here are based on data collected until 31 March 2023, which was considered the end of the second Northern Hemisphere RSV season, because the numbers of reported RSV-ARIs in several Northern Hemisphere countries fell to below the epidemic threshold by that point [16–19]. The current analyses also include data for Southern Hemisphere participants until the same data lock, covering 1 or more Southern Hemisphere season.

The protocol is available as an appendix to Papi et al [14]. Information on ethical conduct is included in the Supplementary Methods.

### Interventions

**Figure 1.**
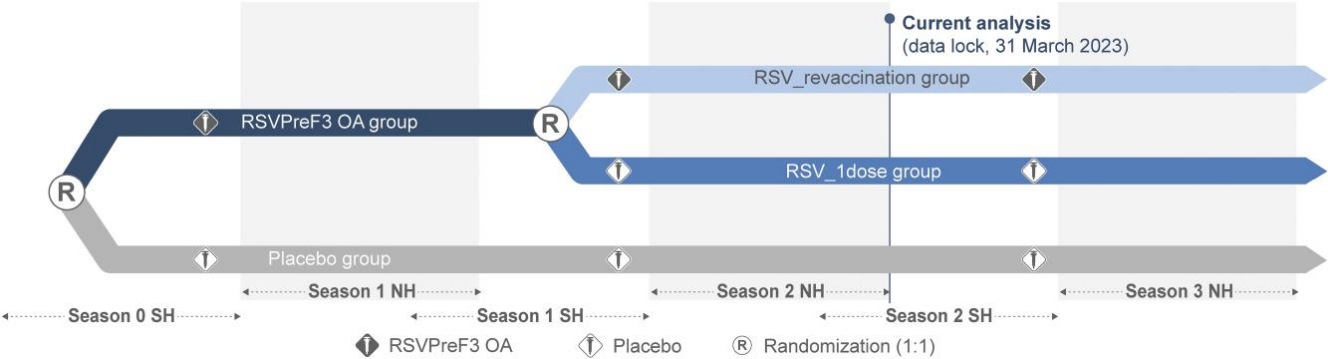
Study design. Participants were randomized before RSV season 1 to receive 1 dose of RSV prefusion F protein–based vaccine (RSVPreF3 OA) or placebo. RSVPreF3 OA recipients were re-randomized before RSV season 2 to receive a second RSVPreF3 OA dose (RSV_revaccination group) or placebo (RSV_1dose group). Participants who received placebo pre–season 1 received a second placebo dose pre–season 2. Abbreviations: NH, Northern Hemisphere; RSV, respiratory syncytial virus; SH, Southern Hemisphere.

Pre–RSV season 1, participants were randomized 1:1 to receive 1 intramuscular injection of AS01_E_-adjuvanted RSVPreF3 OA or placebo. Pre–season 2, RSVPreF3 OA recipients were re-randomized 1:1 to receive a second RSVPreF3 OA dose (RSV_revaccination group) or placebo (RSV_1dose group) (Figure 1). To maintain blinding, participants who had received placebo pre–season 1 received placebo again pre–season 2 (placebo group) (Supplementary Methods). Participants, investigators, and personnel involved in data collection and sample testing were blinded.

### Objectives

Confirmatory secondary objectives were to demonstrate the efficacy of 1 RSVPreF3 OA dose and of a first dose followed by a revaccination dose 1 year later in preventing RSV-LRTD over 2 RSV seasons post–dose 1. Other (descriptive) secondary objectives were to evaluate the efficacy of these 2 regimens in preventing severe RSV-LRTD, RSV-ARI, and RSV-LRTD by RSV subtype, season, year, participant age at dose 1, presence of co-existing medical conditions, and frailty status, and both regimens’ safety.

### Acute Respiratory Illness Surveillance

Acute respiratory illness surveillance was done through spontaneous reporting by the participants and scheduled contacts by the site staff with the participants [14]. Whenever a participant experienced 2 or more ARI symptoms/signs lasting 24 hours or longer, an ARI assessment visit was to occur (Supplementary Methods) [14].

Acute respiratory illness was defined as 2 or more respiratory symptoms/signs or 1 or more respiratory and 1 systemic symptom/sign, lasting 24 hours or longer. Lower respiratory tract disease was defined as 2 or more lower respiratory symptoms/signs (including ≥1 lower respiratory sign) or 3 or more lower respiratory symptoms, lasting 24 hours or longer (Supplementary Methods). The presence of RSV-A/RSV-B was confirmed on nasal self-swabs (collected by the participants) or nasal/throat swabs (collected at the ARI visits) by quantitative reverse transcriptase–polymerase chain reaction (qRT-PCR). An external adjudication committee reviewed all qRT-PCR–confirmed RSV-LRTD cases. Only cases adjudicated as RSV-LRTD by this committee were included in the analyses [14].

### Safety Assessments

Reactogenicity was evaluated in a subset of participants in selected countries/sites (reactogenicity-immunogenicity cohort) who recorded solicited adverse events (AEs) starting within 4 days and unsolicited AEs within 30 days after each vaccine/placebo dose in paper diaries. Participants not part of this cohort recorded all AEs starting within 30 days after each dose (including reactogenicity events) as unsolicited AEs. Serious AEs (SAEs) and potential immune-mediated diseases (pIMDs) were reported for 6 months after each dose; those considered vaccine/placebo-related by the investigator, fatal SAEs, and AEs leading to withdrawal are reported throughout the trial and presented here until the end of season 2.

### Statistical Analyses

We planned to enroll up to 25 000 participants and include approximately1800 participants in the reactogenicity-immunogenicity cohort and assumed a 20% drop-out rate between seasons (Supplementary Methods). Efficacy over 2 seasons of 1 RSVPreF3 OA dose or a first dose followed by revaccination 1 year later (confirmatory secondary objectives) was analyzed in the modified exposed population (participants who received dose 1 and had no RSV-ARI before day 15 post–dose 1). Efficacy over season 2 was analyzed in the dose 2–modified exposed population (participants who received dose 2 and had no RSV-ARI before day 15 post–dose 2).

Confirmatory secondary objectives were met if the lower limits of the 2-sided confidence intervals (CIs) around the efficacy estimates were greater than 20%. A Bonferroni adjustment for multiplicity was applied to evaluate these objectives in parallel; therefore, these analyses were conducted using a 1-sided alpha of 1.25% (97.5% CI). No multiplicity adjustments were applied for the other secondary objectives.

Efficacy against a first occurrence of RSV-LRTD, severe RSV-LRTD, and RSV-ARI over 2 seasons was calculated as 1 minus the relative risk comparing participants receiving 1 RSVPreF3 OA dose or participants receiving the revaccination regimen with participants in the placebo group, using the conditional exact binomial method, based on a Poisson model including season, age, and region as covariates. Season was included as a covariate to account for potential differences between RSV seasons. As studies with other RSV vaccines may not account for these differences, we conducted a post hoc analysis without including season as a covariate.

Periods-at-risk started on day 15 post–dose 1 (or day 15 post–dose 2 for the season 2–only analysis) and ended at the first event, data lock, or drop-out (Supplementary Methods). We also evaluated how efficacy evolved over time by descriptively comparing efficacy over different follow-up times (1 season, 1 year, mid–season 2 [post hoc], 2 seasons) (Supplementary Methods).

Dose 2 reactogenicity was analyzed in the dose 2–solicited safety population (participants in the reactogenicity-immunogenicity cohort with solicited safety data available post–dose 2). Safety was analyzed in the exposed population (participants who received dose 1) or the dose 2–exposed population (participants who received dose 2) (Supplementary Methods).

## RESULTS

### Trial Population

**Figure 2.**
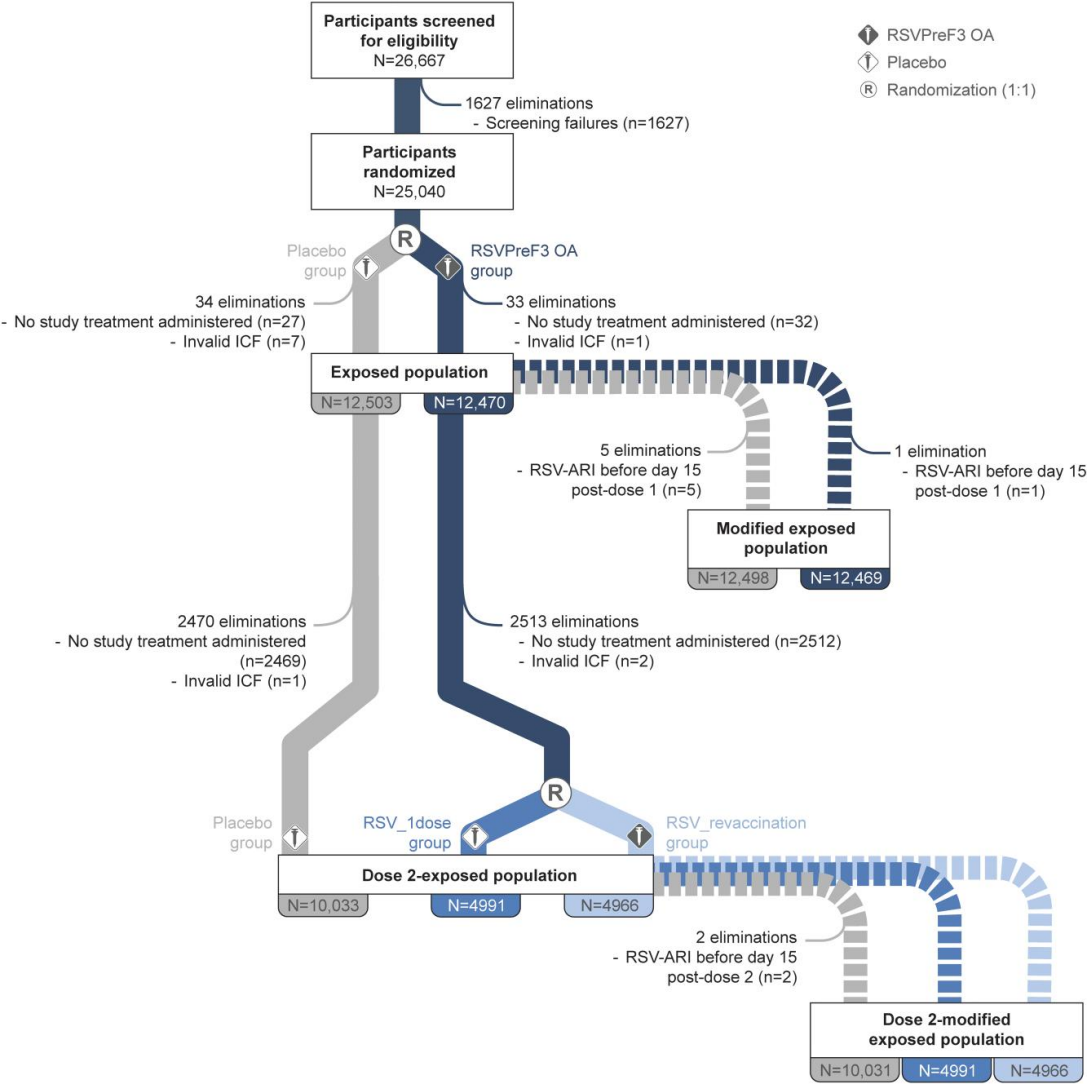
Flow of participants. Abbreviations: ICF, informed consent form; N, number of participants; n, number of participants eliminated; placebo, group of participants randomized to receive placebo pre-season 1 and 2; R, randominzation (1:1); RSV, respiratory syncytial virus; RSV-ARI, RSV-related acute respiratory illness; RSV_1dose, group of participants who received a dose of RSV prefusion F protein-based vaccine (RSVPreF3 OA) pre–season 1 and were randomized to receive a placebo dose pre–season 2; RSVPreF3 OA, group of participants randomized to receive a dose of RSVPreF3 OA pre–RSV season 1; RSV_revaccination, group of participants who received a first dose of RSVPreF3 OA pre–season 1 and were randomized to receive a second RSVPreF3 OA dose (revaccination) pre–season 2.

The exposed population included 24 973 participants, of whom 12 470 received RSVPreF3 OA and 12 503 received placebo pre–season 1. Of these, 19 990 were included in the dose 2–exposed population: 4966 in the RSV_revaccination group, 4991 in the RSV_1dose group, and 10 033 in the placebo group (Figure 2). In total, 4725 (18.9%) participants withdrew before the end of season 2, with similar proportions withdrawn in each group (RSV_revaccination: 19.5%; RSV_1dose: 18.6%; placebo: 18.8%). Baseline characteristics were balanced between groups (Table 1, Supplementary Table 1).

**Table 1.** Baseline Characteristics of the Participants (Exposed Population)

Characteristics	RSVPreF3 OA (N = 12 470)	RSV_revaccination (N = 6242)	RSV_1dose (N = 6228)	Placebo (N = 12 503)
Mean (SD) age, y	69.5 (6.5)	69.6 (6.5)	69.5 (6.4)	69.6 (6.4)
Age group, n (%)				
≥70 y	5507 (44.2)	2759 (44.2)	2748 (44.1)	5521 (44.2)
≥80 y	1018 (8.2)	514 (8.2)	504 (8.1)	1028 (8.2)
60–69 y	6963 (55.8)	3483 (55.8)	3480 (55.9)	6982 (55.8)
70–79 y	4489 (36.0)	2245 (36.0)	2244 (36.0)	4493 (35.9)
Sex, n (%)				
Female	6489 (52.0)	3206 (51.4)	3283 (52.7)	6429 (51.4)
Male	5981 (48.0)	3036 (48.6)	2945 (47.3)	6074 (48.6)
Race, n (%)				
Black	1064 (8.5)	524 (8.4)	540 (8.7)	1101 (8.8)
Asian	953 (7.6)	484 (7.8)	469 (7.5)	956 (7.6)
White	9890 (79.3)	4951 (79.3)	4939 (79.3)	9936 (79.5)
Other	563 (4.5)	283 (4.5)	280 (4.5)	510 (4.1)
Hemisphere,^a^ n (%)				
Northern	11 499 (92.2)	5757 (92.2)	5742 (92.2)	11 526 (92.2)
Southern	971 (7.8)	485 (7.8)	486 (7.8)	977 (7.8)
Type of residence, n (%)				
Community	12 310 (98.7)	6165 (98.8)	6145 (98.7)	12 356 (98.8)
Long-term care facility	160 (1.3)	77 (1.2)	83 (1.3)	147 (1.2)
Mean BMI (SD), kg/m^2^	29.1 (6.1)	29.1 (6.1)	29.2 (6.1)	29.1 (6.0)
Frailty status,^b^ n (%)				
Frail	189 (1.5)	86 (1.4)	103 (1.7)	177 (1.4)
Pre-frail	4795 (38.5)	2437 (39.0)	2358 (37.9)	4782 (38.2)
Fit	7465 (59.9)	3707 (59.4)	3758 (60.3)	7524 (60.2)
Unknown	21 (0.2)	12 (0.2)	9 (0.1)	20 (0.2)
Co-existing conditions of interest,^c^ n (%)				
≥1 condition of interest	4983 (40.0)	2502 (40.1)	2481 (39.8)	4922 (39.4)
Cardiorespiratory condition of interest	2546 (20.4)	1285 (20.6)	1261 (20.2)	2480 (19.8)
Endocrine or metabolic condition of interest	3229 (25.9)	1618 (25.9)	1611 (25.9)	3257 (26.0)

Abbreviations: BMI, body mass index; n (%), number (percentage) of participants in the indicated category; N, number of participants in the exposed population (ie, who received at least dose 1 of RSVPreF3 OA or placebo); placebo, group of participants who received placebo pre–season 1; RSV, respiratory syncytial virus; RSV_1dose, group of participants (subset of RSVPreF3 OA group) who received a dose of RSV prefusion F protein–based vaccine (RSVPreF3 OA) pre–season 1 and were randomized to receive a placebo dose pre–season 2; RSVPreF3 OA, group of participants who received a dose of RSVPreF3 OA pre–RSV season 1; RSV_revaccination, group of participants (subset of RSVPreF3 OA group) who received a first dose of RSVPreF3 OA pre–season 1 and were randomized to receive a second RSVPreF3 OA dose (revaccination) pre–season 2; SD, standard deviation.

^a^Northern Hemisphere countries included in the trial are Belgium, Canada, Estonia, Finland, Germany, Italy, Japan, Mexico, Poland, Republic of Korea, Russian Federation, Spain, United Kingdom, and United States. Southern Hemisphere countries are Australia, New Zealand, and South Africa.

^b^Frailty status was assessed using a gait speed test: frail, participants with a walking speed <0.4 m/s or not able to perform the test; pre-frail, participants with a walking speed of 0.4–0.99 m/s; fit, participants with a walking speed ≥1 m/s.

^c^Conditions of interest included any chronic respiratory/pulmonary disease (including chronic obstructive pulmonary disease and asthma) and chronic heart failure (cardiorespiratory), and diabetes mellitus type 1 or type 2 and advanced liver or renal disease (endocrine or metabolic).

### Efficacy

The modified exposed population included 24 967 participants (RSV_revaccination: 6242; RSV_1dose: 6227; placebo: 12 498). The median efficacy follow-up from day 15 post–dose 1 until the end of RSV season 2 was 17.8 months. During this follow-up, 50 adjudicated RSV-LRTD cases were accrued among participants who received RSVPreF3 OA (10 in the RSVPreF3 OA group during season 1 and 20 cases each in RSV_revaccination and RSV_1dose during season 2), and 139 were accrued in the placebo group (47 in season 1, 92 in season 2). In both seasons, RSV-B was the dominant subtype: 40 of 57 (70.2%) RSV-LRTD cases in season 1 and 83 of 132 (62.9%) in season 2 were RSV-B positive.

#### Efficacy of 1 Dose Over 2 RSV Seasons

**Figure 3.**
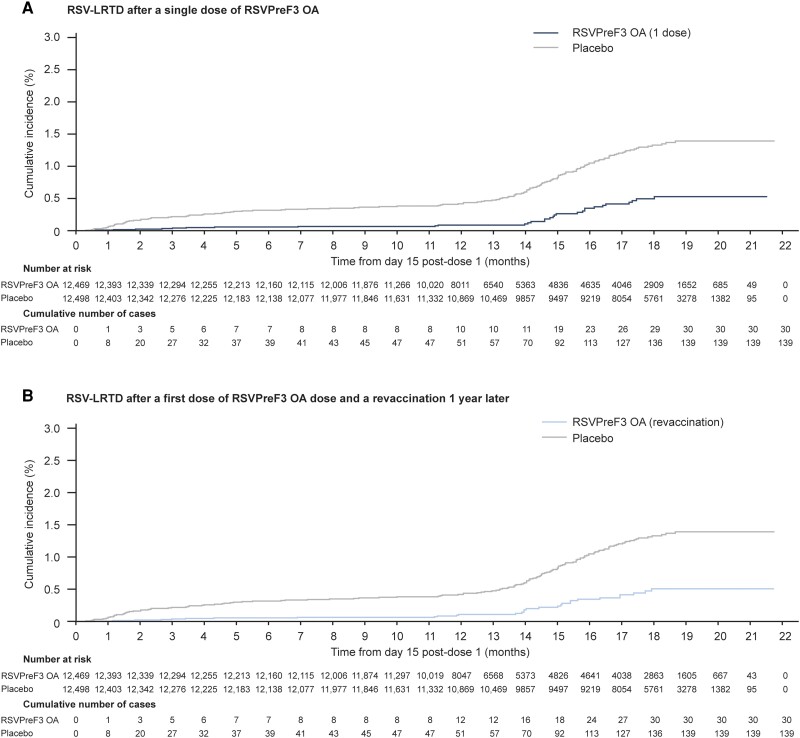
Cumulative incidence of RSV-LRTD over 2 RSV seasons. Analysis includes cases reported from day 15 post–dose 1 until 31 March 2023 for participants who received dose 2 or until 30 September 2022 for those who did not receive dose 2. *A*, For the analysis of a single vaccine dose, participants who received 2 vaccine doses (RSV_revaccination group) contributed to RSV season 1 but were censored before dose 2 administration. *B*, For the analysis of the revaccination regimen, participants who received vaccine as dose 1 and placebo as dose 2 (RSV_1dose group) contributed to season 1 but were censored before dose 2 administration. Abbreviations: RSV, respiratory syncytial virus; RSV-LRTD, RSV-related lower respiratory tract disease confirmed by the adjudication committee; RSVPreF3 OA, RSV prefusion F protein–based vaccine.

The efficacy of 1 RSVPreF3 OA dose given pre–season 1 in preventing RSV-LRTD over 2 full seasons was 67.2% (97.5% CI: 48.2–80.0%; confirmatory objective met) (Table 2). A sensitivity analysis showed similar efficacy against RSV-LRTD without viral coinfections (67.0%; 97.5% CI: 42.9–82.0%). Cumulative incidence curves confirmed sustained efficacy over 2 full seasons (Figure 3*A*). When calculating efficacy without accounting for potential interseason differences (model without season as a covariate), the efficacy over 2 seasons was 74.5% (Table 2).

**Table 2.** Vaccine Efficacy of a Single Dose of RSVPreF3 OA Against a First Occurrence of RSV-LRTD and RSV-ARI Over 2 RSV Seasons (Modified Exposed Population)

	RSVPreF3 OA (1 Dose)				Placebo				Vaccine Efficacy, % (CI^a^)	
Endpoint	N	n	T, person-years	n/T, n/1000 person-years	N	n	T, person-years	n/T, n/1000 person-years	With Season as a Covariate	Without Season as a Covariate
RSV-LRTD										
Overall	12 469	30	14 662.6	2.0	12 498	139	17 269.0	8.0	67.2 (48.2, 80.0)	74.5 (60.0, 84.5)
Severe^b^	12 469	7	14 672.6	0.5	12 498	48	17 320.6	2.8	78.8 (52.6, 92.0)	82.7 (61.6, 93.4)
By subtype^c^										
RSV-A	12 469	6	14 673.7	0.4	12 498	48	17 323.5	2.8	80.5 (54.0, 93.2)	85.2 (65.4, 94.8)
RSV-B	12 469	24	14 665.5	1.6	12 498	90	17 297.6	5.2	59.7 (35.8, 75.5)	68.5 (50.2, 80.8)
By age										
≥70 y	5506	13	6419.5	2.0	5517	65	7614.9	8.5	69.3 (43.4, 84.6)	76.4 (56.7, 88.1)
≥80 y	1017	4	1151.2	3.5	1028	10	1384.4	7.2	38.4 (−118.2, 86.1)	52.6 (−64.2, 89.2)
60–69 y	6963	17	8243.0	2.1	6981	74	9654.1	7.7	65.4 (40.4, 80.9)	72.9 (53.7, 85.0)
70–79 y	4489	9	5268.3	1.7	4489	55	6230.4	8.8	74.9 (48.4, 89.2)	80.7 (60.6, 91.6)
By co-existing condition of interest^d^										
No condition	7486	14	8779.7	1.6	7579	67	10 478.1	6.4	68.3 (42.7, 83.6)	75.0 (55.1, 87.0)
≥1 condition	4983	16	5882.9	2.7	4919	72	6790.9	10.6	66.7 (41.8, 82.0)	74.5 (55.7, 86.1)
≥1 cardiorespiratory condition	2546	10	2997.5	3.3	2479	56	3411.6	16.4	73.8 (47.9, 88.2)	80.1 (60.6, 91.0)
≥1 endocrine or metabolic condition	3229	8	3822.6	2.1	3255	32	4509.4	7.1	63.1 (17.4, 85.4)	70.6 (34.8, 88.3)
By frailty^e^										
Frail	189	2	213.5	9.4	177	1	230.7	4.3	−147.9 (−15 796.3, 88.2)	−116.3 (−12 773.1, 88.9)
Pre-frail	4794	8	5537.3	1.4	4779	47	6478.5	7.3	73.3 (42.4, 89.2)	80.0 (57.3, 91.8)
Fit	7465	20	8891.0	2.2	7522	89	10 533.7	8.4	66.2 (44.3, 80.4)	73.4 (56.5, 84.5)
RSV-ARI										
Overall	12 469	94	14 626.4	6.4	12 498	292	17 167.0	17.0	52.7 (40.0, 63.0)	62.1 (52.1, 70.3)

Analysis includes RSV season 1 data from participants who received dose 1 (vaccine or placebo) and season 2 data from participants who received dose 1 (vaccine or placebo) and dose 2 (placebo). Season 1 data were collected from day 15 post–dose 1 until dose 2 administration or until 30 September 2022 (end of Southern Hemisphere season 1) for participants who did not receive dose 2. Season 2 data were collected from dose 2 until 31 March 2023 (end of Northern Hemisphere season 2). Participants who received vaccine as dose 1 and placebo as dose 2 (RSV_1dose group) contributed to both seasons. Participants who received 2 vaccine doses (RSV_revaccination group) contributed to season 1 but were censored before dose 2 administration. Vaccine efficacy was estimated using a Poisson model adjusted for age, region, and season (“with season as covariate”) or for age and region (“without season as covariate”; post hoc analyses), except for the analysis by age, which used region and season or region only as covariates.

Abbreviations: CI, confidence interval; n, number of participants with ≥1 RSV-LRTD or RSV-ARI; N, number of participants in the modified exposed population; n/T, incidence rate of participants reporting at least 1 event; RSV, respiratory syncytial virus; RSV-ARI, RSV-related acute respiratory illness; RSV-LRTD, RSV-related lower respiratory tract disease confirmed by the adjudication committee; RSVPreF3 OA, RSV prefusion F protein–based vaccine; T, sum of follow-up time (from day 15 post–dose 1 until first occurrence of the event, data lock point, or drop-out).

^a^97.5% CI for confirmatory secondary endpoint (RSV-LRTD, overall); 95% CI for other endpoints.

^b^Severe disease according to either of the 2 case definitions (definition 1 based on clinical signs/investigator assessment or definition 2 based on supportive therapy, see Supplementary Methods). All severe cases met case definition 1; 1 case in the vaccine group and 5 in placebo were confirmed by the adjudication committee as also meeting case definition 2.

^c^RSV subtype was unknown for 1 RSV-LRTD case (placebo group).

^d^Conditions of interest are as explained in Table 1, footnote c.

^e^Frailty status was assessed using a gait speed test as explained in Table 1, footnote b.

The efficacy over 2 seasons was 78.8% against severe RSV-LRTD and 52.7% against RSV-ARI. Efficacy was observed against medically attended RSV-LRTD and RSV-ARI (Supplementary Table 2) but could not be evaluated against RSV-related hospitalizations, because only 1 participant in the RSV_1dose group and 5 in the placebo group were hospitalized for RSV-related respiratory disease.

Sustained efficacy over 2 seasons was observed against RSV-LRTD caused by RSV-A (80.5%) and RSV-B (59.7%), among participants 60–69 years old (65.4%) and 70–79 years old (74.9%), those with 1 or more co-existing condition of interest (66.7%), and pre-frail participants (73.3%) (Table 2). Too few RSV-LRTD cases were reported among participants aged 80 years and older and frail participants to conclude on efficacy in these subgroups.

Although efficacy against RSV-LRTD remained high during 2 seasons, estimates tended to decline with increasing follow-up postvaccination: 82.6% over 1 season (median follow-up, 6.7 months) [14], 78.9% over 1 year, 77.3% until mid–season 2 (median follow-up, 13.9 months), and 67.2% over 2 full seasons (median follow-up, 17.8 months). Similar trends were observed for severe RSV-LRTD and RSV-ARI (Table 3).

**Table 3.** Vaccine Efficacy of a Single Dose of RSVPreF3 OA Against a First Occurrence of RSV-LRTD and RSV-ARI After Different Follow-up Times Postvaccination (Modified Exposed Population)

Follow-up Postvaccination	RSV-LRTD, % (CI^a^)		Severe RSV-LRTD,^b^ % (CI^a^)		RSV-ARI, % (CI^a^)	
	With Season as a Covariate	Without Season as a Covariate	With Season as a Covariate	Without Season as a Covariate	With Season as a Covariate	Without Season as a Covariate
Season 1 (6.7 mo)	NA	82.6 (57.9, 94.1)	NA	94.1 (62.4, 99.9)	NA	71.7 (56.2, 82.3)
1 y (12 mo)	NA	78.9 (57.6, 90.5)	NA	ND	NA	ND
Season 1 + mid-season 2 (13.9 mo)	77.3 (60.2, 87.9)	80.9 (66.7, 89.8)	84.6 (56.4, 96.1)	86.8 (63.0, 96.6)	56.5 (41.6, 68.0)	62.8 (50.3, 72.6)
Season 1 + 2 (17.8 mo)	67.2 (48.2, 80.0)	74.5 (60.0, 84.5)	78.8 (52.6, 92.0)	82.7 (61.6, 93.4)	52.7 (40.0, 63.0)	62.1 (52.1, 70.3)

“Season 1” follow-up included cases collected from day 15 post–dose 1 until 11 April 2022 (end of Northern Hemisphere season 1). The “1 year” follow-up included cases collected from day 15 post–dose 1 until dose 2 administration or until 30 September 2022 (end of Southern Hemisphere season 1) if no dose 2 was administered. “Season 1 + mid-season 2” follow-up included cases collected from day 15 post–dose 1 until dose 2 administration or until 30 September 2022 (end of Southern Hemisphere season 1) if no dose 2 was administered and cases collected from dose 2 until 30 November 2022 (mid–Northern Hemisphere season 2). “Season 1 + 2” follow-up included cases collected from day 15 post–dose 1 until dose 2 administration or until 30 September 2022 (end of Southern Hemisphere season 1) if no dose 2 was administered and cases collected from dose 2 until 31 March 2023 (end of Northern Hemisphere season 2). Vaccine efficacy was estimated using a Poisson model adjusted for age, region, and season (“with season as a covariate”) or for age and region (“without season as a covariate”; post hoc analyses).

Abbreviations: CI, confidence interval; NA, not applicable; ND, not determined; RSV, respiratory syncytial virus; RSV-ARI, RSV-related acute respiratory illness; RSV-LRTD, RSV-related lower respiratory tract disease confirmed by the adjudication committee; RSVPreF3 OA, RSV prefusion F protein–based vaccine.

^a^96.95% CI for primary endpoint (RSV-LRTD, season 1); 97.5% CI for confirmatory secondary endpoint (RSV-LRTD, season 1 + 2); 95% CI for other endpoints.

^b^Severe disease according to either of the 2 case definitions (definition 1 based on clinical signs/investigator assessment or definition 2 based on supportive therapy, see Supplementary Methods).

When considering season 2 only, the efficacy of 1 RSVPreF3 OA dose given pre–season 1 was 56.1% (95% CI: 28.2–74.4%) against RSV-LRTD, 64.2% (95% CI: 6.2–89.2%) against severe RSV-LRTD, and 40.6% (95% CI: 19.0–57.0%) against RSV-ARI over a median follow-up of 6.3 months during season 2 (Supplementary Table 3).

#### Efficacy of a First Dose Followed by Revaccination 1 Year Later

The efficacy of a first RSVPreF3 OA dose given pre–season 1 and revaccination given pre–season 2 in preventing a first occurrence of RSV-LRTD over 2 seasons post–dose 1 was 67.1% (97.5% CI: 48.1–80.0%; confirmatory objective met) (Table 4). Cumulative incidence curves confirmed the efficacy of the revaccination regimen over 2 seasons (Figure 3*B*).

**Table 4.** Vaccine Efficacy of a First RSVPreF3 OA Dose Followed by Revaccination 1 Year Later Against a First Occurrence of RSV-LRTD and RSV-ARI Over 2 RSV Seasons Post–Dose 1 (Modified Exposed Population)

	RSVPreF3 OA (revaccination)				Placebo				Vaccine Efficacy With Season as a Covariate, % (CI^a^)
Endpoint	N	n	T, person-years	n/T, n/1000 person-years	N	n	T, person-years	n/T, n/1000 person-years	
RSV-LRTD									
Overall	12 469	30	14 660.5	2.0	12 498	139	17 269.0	8.0	67.1 (48.1, 80.0)
Severe^b^	12 469	7	14 672.9	0.5	12 498	48	17 320.6	2.8	78.8 (52.5, 92.0)
By subtype^c^									
RSV-A	12 469	13	14 670.0	0.9	12 498	48	17 323.5	2.8	55.9 (16.8, 78.2)
RSV-B	12 469	17	14 666.1	1.2	12 498	90	17 297.6	5.2	72.1 (52.5, 84.5)
By age									
≥70 y	5506	16	6434.8	2.5	5517	65	7614.9	8.5	61.9 (33.0, 79.6)
≥80 y	1017	4	1153.9	3.5	1028	10	1384.4	7.2	38.6 (−117.2, 86.2)
60–69 y	6963	14	8225.6	1.7	6981	74	9654.1	7.7	71.6 (48.9, 85.2)
70–79 y	4489	12	5280.9	2.3	4489	55	6230.4	8.8	66.2 (35.7, 83.6)
By co-existing condition of interest^d^									
No condition	7486	18	8788.0	2.0	7579	67	10 478.1	6.4	58.5 (28.8, 76.9)
≥1 condition	4983	12	5872.5	2.0	4919	72	6790.9	10.6	75.1 (53.6, 87.8)
≥1 cardiorespiratory condition	2546	7	2987.5	2.3	2479	56	3411.6	16.4	81.3 (58.6, 92.9)
≥1 endocrine or metabolic condition	3229	7	3814.7	1.8	3255	32	4509.4	7.1	67.5 (24.2, 88.0)
By frailty^e^									
Frail	189	1	209.4	4.8	177	1	230.7	4.3	13.6 (−6751.4, 98.9)
Pre-frail	4794	7	5561.0	1.3	4779	47	6478.5	7.3	77.3 (49.1, 91.4)
Fit	7465	22	8867.6	2.5	7522	89	10 533.7	8.4	62.2 (38.8, 77.5)
RSV-ARI									
Overall	12 469	80	14 630.1	5.5	12 498	292	17 167.0	17.0	60.3 (48.8, 69.5)

Analysis includes RSV season 1 data from participants who received dose 1 (vaccine or placebo) and season 2 data from participants who received dose 1 and dose 2 (2 vaccine doses or 2 placebo doses). Season 1 data were collected from day 15 post–dose 1 until dose 2 administration or until 30 September 2022 (end of Southern Hemisphere season 1) for participants who did not receive dose 2. Season 2 data were collected from dose 2 until 31 March 2023 (end of Northern Hemisphere season 2). Participants who received vaccine as dose 1 and placebo as dose 2 (RSV_1dose group) contributed to season 1 but were censored before dose 2 administration. Participants who received 2 vaccine doses (RSV_revaccination group) contributed to both seasons. Vaccine efficacy was estimated using a Poisson model adjusted for age, region, and season, except for the analysis by age, which used region and season as covariates.

Abbreviations: CI, confidence interval; n, number of participants with ≥1 RSV-LRTD or RSV-ARI; N, number of participants in the modified exposed population; n/T, incidence rate of participants reporting at least 1 event; RSV, respiratory syncytial virus; RSV-ARI, RSV-related acute respiratory illness; RSV-LRTD, RSV-related lower respiratory tract disease confirmed by the adjudication committee; RSVPreF3 OA, RSV prefusion F protein–based vaccine; T, sum of follow-up time (from day 15 post–dose 1 until first occurrence of the event, data lock point, or drop-out).

^a^97.5% CI for confirmatory secondary endpoint (RSV-LRTD, overall); 95% CI for other endpoints.

^b^Severe disease according to either of the 2 case definitions (definition 1 based on clinical signs/investigator assessment or definition 2 based on supportive therapy; see Supplementary Methods). All severe cases met case definition 1; no cases in the vaccine group and 5 in placebo were confirmed by the adjudication committee as also meeting case definition 2.

^c^RSV subtype was unknown for 1 RSV-LRTD case (placebo group).

^d^Conditions of interest are as explained in Table 1, footnote c.

^e^Frailty status was assessed using a gait speed test as explained in Table 1, footnote b.

The efficacy of the revaccination regimen over 2 seasons was 78.8% against severe RSV-LRTD and 60.3% against RSV-ARI. None of the participants who received RSVPreF3 OA revaccination were hospitalized due to RSV-related respiratory disease.

The revaccination regimen was efficacious over 2 seasons against RSV-LRTD caused by RSV-A (55.9%) and RSV-B (72.1%) among participants aged 60–69 years old (71.6%) and 70–79 years old (66.2%), those with 1 or more co-existing condition of interest (75.1%), and pre-frail participants (77.3%) (Table 4). We could not conclude on efficacy in participants aged 80 years and older and frail participants due to too few cases.

The efficacy of the revaccination regimen over season 2 only was 55.9% (95% CI: 27.9–74.3%) against RSV-LRTD, 64.1% (95% CI: 5.9–89.2%) against severe RSV-LRTD, and 55.8% (95% CI: 37.5–69.5%) against RSV-ARI (median follow-up, 6.3 months during season 2) (Supplementary Table 3).

### Safety

**Figure 4.**
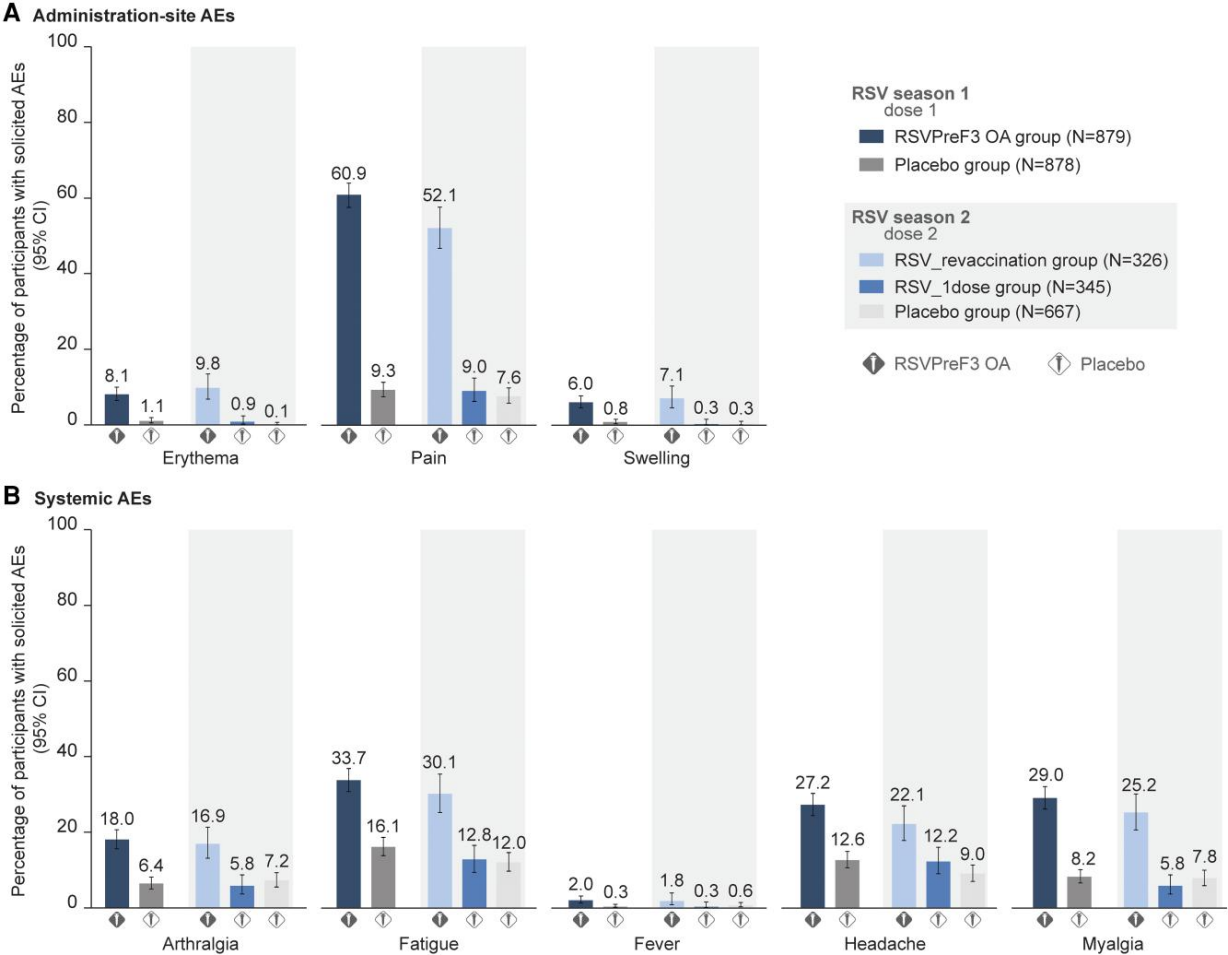
Solicited AEs after a first or second dose of RSVPreF3 OA or placebo. AE rates after dose 1 (RSV season 1) were analyzed in the solicited safety population; AE rates after dose 2 (RSV season 2) were analyzed in the dose 2–solicited safety population. Fever was defined as a temperature ≥38.0°C by any route. Abbreviations: AE, adverse event; CI, confidence interval (depicted as error bars); N, maximum number of participants with solicited safety data available; placebo group, group of participants who received placebo pre–season 1 and pre–season 2; RSV, respiratory syncytial virus; RSV_1dose group, group of participants who received a single RSV prefusion F protein–based vaccine (RSVPreF3) OA dose pre–season 1 and a placebo dose pre–season 2; RSVPreF3 OA group, group of participants who received a dose of RSVPreF3 OA pre–season 1; RSV_revaccination group, group of participants who received a first RSVPreF3 OA dose pre–season 1 and a second RSVPreF3 OA dose (revaccination) pre–season 2.

In the dose 2–solicited safety population, 63.6% of participants in the RSV_revaccination group reported solicited AEs within 4 days after the second RSVPreF3 OA dose, versus 26.4% and 22.0% after the placebo doses in the RSV_1dose and placebo groups, respectively (Table 5). Pain and fatigue were the most frequently reported solicited administration-site and systemic AEs (Figure 4). Most events were mild or moderate and resolved within 2–3 days. Solicited AE rates after RSVPreF3 OA dose 2 were similar to those post–dose 1 (Figure 4).

**Table 5.** Solicited and Unsolicited Adverse Events After a Second RSVPreF3 OA Dose or Placebo

Adverse Event	RSV_revaccination		RSV_1dose		Placebo	
	n	% (95% CI)	n	% (95% CI)	n	% (95% CI)
Dose 2–solicited safety population	N = 327		N = 345		N = 667	
Solicited AEs (within 4 d post–dose 2)						
Any solicited AE	208	63.6 (58.1, 68.8)	91	26.4 (21.8, 31.4)	147	22.0 (18.9, 25.4)
Grade 3	15	4.6 (2.6, 7.5)	3	0.9 (.2, 2.5)	10	1.5 (.7, 2.7)
Solicited administration-site AE	177	54.1 (48.6, 59.6)	33	9.6 (6.7, 13.2)	51	7.6 (5.7, 9.9)
Grade 3	*10*	…	*10*	…	*10*	…
Solicited systemic AE	146	44.6 (39.2, 50.2)	74	21.4 (17.2, 26.2)	121	18.1 (15.3, 21.3)
Grade 3	6	1.8 (.7, 4.0)	3	0.9 (.2, 2.5)	10	1.5 (.7, 2.7)
Unsolicited AEs (within 30 d post-dose 2)						
Any unsolicited AE	51	15.6 (11.8, 20.0)	36	10.4 (7.4, 14.2)	71	10.6 (8.4, 13.2)
Grade 3	9	2.8 (1.3, 5.2)	7	2.0 (.8, 4.1)	9	1.3 (.6, 2.5)
Dose 2–exposed population	N = 4966		N = 4991		N = 10 033	
Unsolicited AEs (within 30 d post–dose 2)^a^						
Any unsolicited AE	1416	28.5 (27.3, 29.8)	791	15.8 (14.8, 16.9)	1495	14.9 (14.2, 15.6)
Grade 3	107	2.2 (1.8, 2.6)	54	1.1 (.8, 1.4)	120	1.2 (1.0, 1.4)
Any related unsolicited AE	947	19.1 (18.0, 20.2)	215	4.3 (3.8, 4.9)	326	3.2 (2.9, 3.6)
Grade 3	56	1.1 (.9, 1.5)	7	0.1 (.1, .3)	12	0.1 (.1, .2)
Any medically attended unsolicited AE	337	6.8 (6.1, 7.5)	306	6.1 (5.5, 6.8)	630	6.3 (5.8, 6.8)
Serious AEs and potential immune-mediated diseases (within 6 mo post–dose 2)						
Any serious AE	210	4.2 (3.7, 4.8)	219	4.4 (3.8, 5.0)	461	4.6 (4.2, 5.0)
Any potential immune-mediated disease	14	0.3 (.2, .5)	19	0.4 (.2, .6)	35	0.3 (.2, .5)
Serious AEs and potential immune-mediated diseases (from dose 2 up to data lock point)						
Any related serious AE^b^	4	0.1 (.0, .2)	2	0.0 (.0, .1)	4	0.0 (.0, .1)
Any related potential immune-mediated disease^b^	1	0.0 (.0, .1)	1	0.0 (.0, .1)	2	0.0 (.0, .1)
Any fatal serious AE	20	0.4 (.2, .6)	26	0.5 (.3, .8)	41	0.4 (.3, .6)

“Grade 3” was defined as diameter >100 mm for erythema and swelling, temperature >39.0°C for fever, preventing normal everyday activities for all other events; “related” indicates considered related to vaccine/placebo administration by investigator assessment.

Abbreviations: AE, adverse event; CI, confidence interval; n/%, number/percentage of participants reporting the event at least once (numbers between asterisks, eg, *10, * indicate that data by group are blinded to avoid participant-level unblinding of the study team; the number between the asterisks shows the total number across the 3 groups); N, number of participants in the indicated analysis population; placebo, group of participants who received placebo pre–season 1 and pre–season 2; RSV, respiratory syncytial virus; RSV_1dose, group of participants who received a single RSV prefusion F protein–based vaccine (RSVPreF3 OA) dose pre–season 1 and a placebo dose pre–season 2; RSV_revaccination, group of participants who received a first dose of RSVPreF3 OA pre–season 1 and a second RSVPreF3 OA dose (revaccination) pre–season 2.

^a^Most unsolicited adverse events in the RSV_revaccination group were reactogenicity events, primarily in participants who were not included in the reactogenicity-immunogenicity cohort and thus reported reactogenicity events as unsolicited AEs.

^b^Related serious AEs and potential immune-mediated disease are detailed in the Supplementary Results.

In the dose 2–exposed population, 28.5% (RSV_revaccination), 15.8% (RSV_1dose), and 14.9% (placebo) of participants reported unsolicited AEs within 30 days post–dose 2 (Table 5). The higher AE rate in the RSV_revaccination group is primarily due to reactogenicity events, reported as unsolicited AEs by participants who were not included in the dose 2–solicited safety population.

Both SAEs and pIMDs were reported at similar rates in the 3 groups (Table 5). The most common system organ classes were cardiac disorders and infections for SAEs (Supplementary Table 4) and musculoskeletal/connective tissue disorders and skin/subcutaneous tissue disorders for pIMDs (Supplementary Table 5). No cases of acute disseminated encephalomyelitis, Guillain-Barré syndrome, or other demyelinating disorders were reported.

A numerical imbalance was noted in atrial fibrillation within 30 days post–dose 1 (higher rates in vaccine than placebo recipients) but not within 30 days post–dose 2. Within 6 months postvaccination, a numerical imbalance in atrial fibrillation reported as an SAE was seen post–dose 2 but not post–dose 1 (Supplementary Results). In-depth review of atrial fibrillation cases did not support a vaccine-related effect; the data more plausibly reflect a typical picture of paroxysms of atrial fibrillation in a small number of high-risk individuals, at a rate no higher than expected in this population.

From dose 2 until the end of season 2, 20 (0.4%) participants in the RSV_ revaccination group, 26 (0.5%) in the RSV_1dose group, and 41 (0.4%) in the placebo group died, with general disorders and cardiac disorders as most common system organ classes (Supplementary Table 6).

During the entire follow-up period from dose 1 until the end of season 2, 297 participants died: 77 (1.2%) in the RSV_revaccination group, 71 (1.1%) in the RSV_1dose group, and 149 (1.2%) in the placebo group. Four fatal SAEs (group allocation still blinded) were considered by the investigator as possibly vaccine/placebo-related; 3 of these (pulmonary embolism 148 days post–dose 1, cardiopulmonary failure 31 days post–dose 1, and unknown cause 326 days post–dose 1) occurred between doses 1 and 2 (see [14]); 1 occurred post–dose 2 (myocardial ischemia 37 days post–dose 2). Because pre-existing and concurrent medical conditions were present in these participants, the sponsor considered alternative explanations for these deaths plausible.

## DISCUSSION

**Figure 5.**
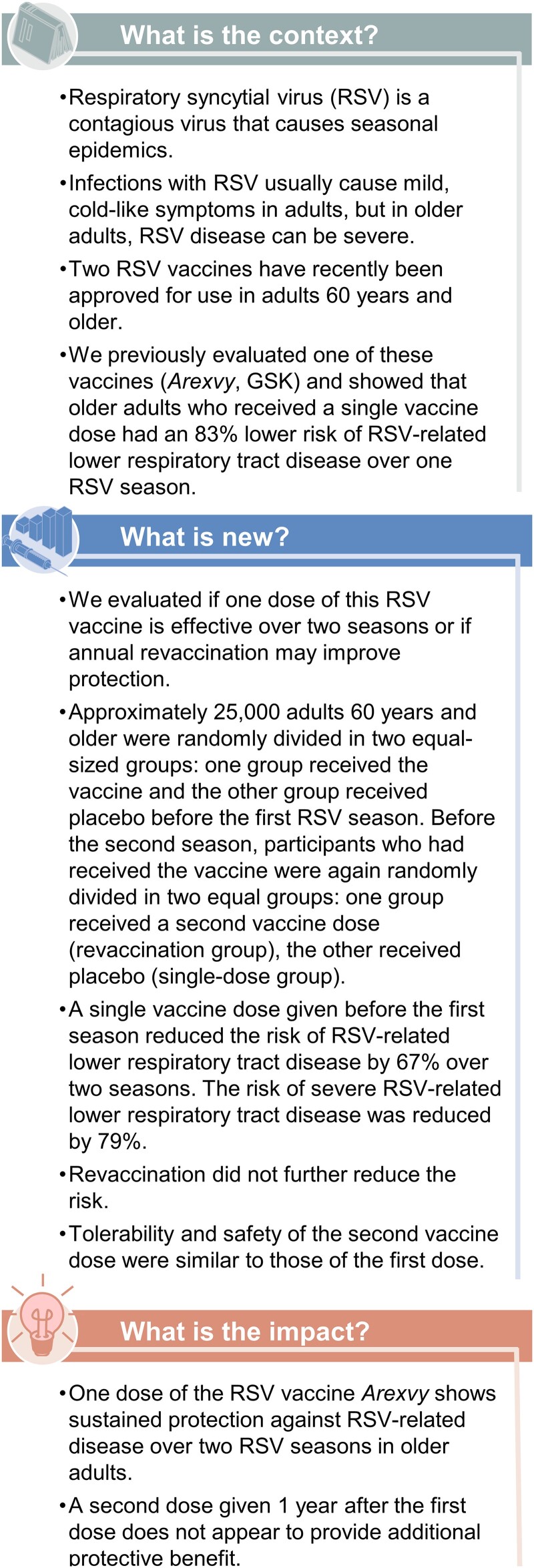
Plain-language summary.

This trial demonstrated that 1 RSVPreF3 OA dose was efficacious in preventing RSV-related disease in participants aged60 years and older over 2 full RSV seasons, with efficacy estimates of 67.2% against RSV-LRTD, 78.8% against severe RSV-LRTD, and 52.7% against RSV-ARI. These estimates were similar to those in participants who received RSVPreF3 OA revaccination pre–season 2 (67.1% against RSV-LRTD, 78.8% against severe RSV-LRTD, and 60.3% against RSV-ARI over 2 seasons post–dose 1). Hence, RSVPreF3 OA revaccination given 1 year post–dose 1 did not seem to provide additional efficacy benefit in the overall study population. Findings are summarized in plain language in Figure 5. Sustained efficacy of 1 RSVPreF3 OA dose over 2 seasons was observed with advanced age, among pre-frail participants, and those with underlying conditions of interest.

Our prespecified efficacy analysis over 2 RSV seasons was based on a model including season as a covariate to account for differences between seasons in RSV-LRTD incidence rates, efficacy, follow-up time, or group sizes. Studies with other RSV vaccines may not account for interseason differences. Therefore, we performed a post hoc analysis using the model without season as a covariate. In this post hoc analysis, efficacy is driven by the season with the largest person-years of follow-up (ie, season 1). Hence, efficacy estimates tended to be higher than in our prespecified analysis.

Although no immunological correlate of protection against RSV disease has been established, the perceived lack of additional efficacy benefit of revaccination given 1 year post–dose 1 is in line with results from immunogenicity studies showing that a second dose of RSVPreF3 OA or other RSV prefusion F–based vaccines given 1 year post–dose 1 induced a humoral immune response, but post–dose 2 RSV neutralizing titers or immunoglobulin G concentrations did not reach post–dose 1 levels [20–24]. Similarly, RSVPreF3 OA revaccination given 18 months after 2 initial RSVPreF3 OA doses boosted RSV neutralizing titers, but post-revaccination titers were lower than post–dose 2 titers [25]. The limited booster effect of revaccination given 12–18 months after initial vaccination might relate to the high levels of RSV-specific antibodies persisting up to that point [20–25], which may also explain the sustained efficacy over 2 seasons. Further research is needed to evaluate whether efficacy is maintained over more than 2 seasons and if extending the interval between initial vaccination and revaccination may enhance boosting. Longer intervals between vaccine doses have been associated with stronger immune responses and improved protective efficacy for other vaccines [26–28].

Epidemiological differences between the 2 RSV seasons in our trial may have contributed to the lower efficacy over 2 seasons than over the first season [14]. Overall, in our study, RSV-B was the dominant subtype in both seasons, although both subtypes were shown to co-circulate in various proportions worldwide depending on the region during these seasons [29–34]. During season 2, there were more RSV circulation and RSV disease in the community than during season 1 [16, 18], presumably because public health measures to reduce severe acute respiratory syndrome coronavirus 2 (SARS-CoV-2) spread had been lifted in many countries. Other respiratory viruses also circulated more during season 2 than season 1 in some regions [17, 35]. Despite this higher circulation and a larger proportion of viral coinfections during season 2 in our trial (Supplementary Results), a sensitivity analysis showed that efficacy was not impacted by the presence of viral coinfections.

The high efficacy of RSVPreF3 OA observed over 1 year (78.9% against RSV-LRTD) suggests that RSVPreF3 OA may be administered several months before the start of the RSV season without losing substantial clinical benefit throughout the season. This, together with results of studies showing acceptable reactogenicity/safety and no clinically relevant immunological interference of co-administration with influenza vaccines [36, 37], supports flexible administration of RSVPreF3 OA, either concomitantly with other vaccines (eg, influenza) at the start of the season or separately ahead of the season.

The reactogenicity/safety profile of the RSVPreF3 OA revaccination dose was comparable to that of the first dose [14], with mostly mild-to-moderate and transient adverse reactions. Safety follow-up for approximately 18 months post–dose 1 confirmed the vaccine's acceptable safety profile.

As described previously [14], a limitation of our trial was the relatively low number of frail participants and participants aged 80 years and older and the low number of RSV-LRTD cases in these subgroups at high risk of severe RSV disease. Conclusions on efficacy in these subgroups might be drawn as more cases accumulate during the trial. Results from another RSVPreF3 OA phase 3 study (NCT04732871) showed RSV neutralizing titers at 12 months postvaccination in individuals aged 80 years and older similar to those in individuals aged 60–69 and 70–79 years [38]. The number of RSV-related hospitalizations was low in all arms; therefore, longer follow-up and data from larger populations are needed to inform the efficacy against RSV-related hospitalizations. Further, the comparison of efficacy between the 2 seasons is complicated by the sizes of the RSV_revaccination and RSV_1dose groups at season 2, which were half that of the RSVPreF3 OA group at season 1.

Our trial showed that 1 dose of RSVPreF3 OA had an acceptable safety profile and was efficacious against RSV-LRTD during at least 2 RSV seasons in individuals aged 60 years and older, including those with an increased risk of severe RSV disease. Revaccination 1 year post–dose 1 was well tolerated but did not increase efficacy over 2 seasons combined or over season 2 alone. Further research is needed to determine the optimal timing of revaccination.

## Supplementary Data

Supplementary materials are available at *Clinical Infectious Diseases* online. Consisting of data provided by the authors to benefit the reader, the posted materials are not copyedited and are the sole responsibility of the authors, so questions or comments should be addressed to the corresponding author.

## Supplementary Material

ciae010_Supplementary_Data

## Notes

***Author Contributions.*** C. V., N. D., O. G., L. F., M.-P. D., L. K., V. H., A. O., M. V. d. W., and D. D. were involved in the conception or design of the study/project. M. G. I., A. P., E. A., R. G. F., J. M. L., D.-G. L., I. L.-R., F. M.-T., T. F. S., and R. N. v. Z.-S. participated in the acquisition or generation of the data. O. G., L. F., and M.-P. D. performed the data analysis. M. G. I., A. P., E. A., R. G. F., J. M. L., D.-G. L., I. L.-R., F. M.-T., T. F. S., R. N. v. Z.-S., C. V., N. D., L. F., M.-P. D., A. O., M. V. d. W., and D. D. were involved in the interpretation of the data.

***Acknowledgments.*** The authors are grateful to all participants, study team members, investigators, and study site staff for their contributions to the study. They also thank Akkodis Belgium (c/o GSK) for medical writing (by Natalie Denef), design, and publication coordination support.

***Data-sharing statement.*** Anonymized data can be requested from www.clinicalstudydatarequest.com after completion of the trial.

***Trademarks.*** Arexvy and AS01_E_ are trademarks licensed to or owned by GSK.

***AReSVi-006 Study Group members (listed alphabetically)*.** Adams, Mark; Adams, Michael; Agutu, Clara; Akite, Elaine Jacqueline; Alt, Ingrid; Andrews, Charles; Antonelli-Incalzi, Rafaelle; Asatryan, Asmik; Bahrami, Ghazaleh; Bargagli, Elena; Bhorat, Qasim; Bird, Paul; Borowy, Przemyslaw; Boutry, Celine; Brotons Cuixart, Carles; Browder, David; Brown, Judith; Buntinx, Erik; Cameron, Donald; Campora, Laura; Cartier, Cyrille; Chinsky, Kenneth; Choi, Melissa; Choo, Eun-Ju; Collete, Delphine; Corral Carrillo, Maria; Cuadripani, Susanna; Davis, Matthew G; de Heusch, Magali; de Looze, Ferdinandus; De Meulemeester, Marc; De Negri, Ferdinando; de Schrevel, Nathalie; DeAtkine, David; Dedkova, Viktoriya; Dzongowski, Peter; Eckermann, Tamara; Essink, Brandon; Faulkner, Karen; Ferguson, Murdo; Fuller, Gregory; Galan Melendez, Isabel Maria; Gentile, Ivan; Ghesquiere, Wayne; Grimard, Doria; Halperin, Scott; Heer, Amardeep; Helman, Laura; Hotermans, Andre; Jelinek, Tomas; Kamerbeek, Jackie; Kim, Hyo Youl; Kimmel, Murray; Koch, Mark; Kokko, Satu; Koski, Susanna; Kotb, Shady; Lalueza, Antonio; Lee, Jin-Soo; Lins, Muriel; Lombaard, Johannes; Mahomed, Akbar; Malerba, Mario; Marechal, Celine; Marion, Sandie; Martinot, Jean-Benoit; Masuet-Aumatell, Cristina; McNally, Damien; Medina Pech, Carlos Eduardo; Mendez Galvan, Jorge; Mercati, Lise; Mesaros, Narcisa Elena; Mesotten, Dieter; Mitha, Essack; Mngadi, Kathryn; Moeckesch, Beate; Montgomery, Barnaby; Murray, Linda; Nally, Rhiannon; Narejos Perez, Silvia; Newberg, Joseph; Nugent, Paul; Ochoa Mazarro, Dolores; Oda, Harunori; Orso, Maurizio; Ortiz Molina, Jacinto; Pak, Tatiana; Park, Dae Won; Patel, Meenakshi; Patel, Minesh; Pedro Pijoan, Anna Maria; Perez, Alberto Borobia; Perez-Breva, Lina; Perez Vera, Merce; Pileggi, Claudia; Pregliasco, Fabrizio; Pretswell, Carol; Quinn, Dean; Reynolds, Michele; Romanenko, Viktor; Rosen, Jeffrey; Roy, Nathalie; Ruiz Antoran, Belen; Sahakyan, Vardine; Sakata, Hideaki; Sauter, Joachim; Schaefer, Axel; Sein Anand, Izabela; Serra Rexach, Jose Antonio; Shu, David; Siig, Andres; Simon, William; Smakotina, Svetlana; Steenackers, Katie; Stephan, Brigitte; Tafuri, Silvio; Takazawa, Kenji; Tellier, Guy; Terryn, Wim; Tharenos, Leslie; Thomas, Nick; Toursarkissian, Nicole; Ukkonen, Benita; Vale, Noah; Van Landegem, Pieter-Jan; Vanden Abeele, Carline; Vermeersch, Lode; Vitale, Francesco; Voloshyna, Olga; White, Judith; Wie, Seong-Heon; Wilson, Jonathan; Ylisastigui, Pedro; Zocco, Manuel.

***Financial support*.** This work was supported by GlaxoSmithKline Biologicals SA, which funded this trial, was involved in all stages of trial conduct, and took charge of all costs associated with the development and publication of this manuscript.
