# Supplementary material for: Efficacy and Safety of Respiratory Syncytial Virus (RSV) Prefusion F Protein Vaccine (RSVPreF3 OA) in Older Adults Over 2 RSV Seasons
Source: Clin Infect Dis. 2024 Jan 22;78(6):1732–44. doi: 10.1093/cid/ciae010 (PMC11175669; doi:10.1093/cid/ciae010)
Supplement: ciae010_Supplementary_Data [file ciae010_supplementary_data.docx]

**Supplementary material**

**Table of Contents**

[Supplementary methods 3](#_Toc153540699)

[Ethical conduct 3](#_Toc153540700)

[Vaccine and placebo composition 3](#_Toc153540701)

[Randomization 3](#_Toc153540702)

[Acute respiratory illness (ARI) surveillance 4](#_Toc153540703)

[Case definitions 4](#_Toc153540704)

[Statistical analyses 8](#_Toc153540705)

[Supplementary results 12](#_Toc153540706)

[Viral co-infections 12](#_Toc153540707)

[Safety 12](#_Toc153540708)

[Supplementary tables 14](#_Toc153540709)

[Table S1. Baseline characteristics of the participants (dose 2-exposed population) 14](#_Toc153540710)

[Table S2. Vaccine efficacy of a single dose of RSVPreF3 OA against first occurrence of medically attended RSV-LRTD and RSV-ARI over 2 RSV seasons (modified exposed population) 17](#_Toc153540711)

[Table S3. Vaccine efficacy of a single dose of RSVPreF3 OA and of a first RSVPreF3 OA dose followed by revaccination 1 year later, against first occurrence of RSV-LRTD and RSV-ARI during RSV season 2 only (dose 2-modified exposed population) 18](#_Toc153540712)

[Table S4. Most common^a^ serious adverse events within 6 months post-dose 2, classified by MedDRA primary system organ class and high-level term (dose 2-exposed population) 23](#_Toc153540713)

[Table S5. Most common^a^ potential immune-mediated diseases with onset within 6 months post-dose 2, classified by MedDRA primary system organ class and high-level term (dose 2-exposed population) 27](#_Toc153540714)

[Table S6. Most common^a^ fatal serious adverse events from dose 2 administration until the end of season 2, classified by MedDRA primary system organ class and high-level term (dose 2-exposed population) 29](#_Toc153540715)

[Supplementary references 31](#_Toc153540716)

Supplementary methods

Ethical conduct

The protocol, amendments, and other trial-related documents were approved by the trial sites’ independent ethics committees. The trial is conducted according to the Declaration of Helsinki, the Council for International Organizations of Medical Sciences, Good Clinical Practice, and local laws and regulations. All participants gave written or witnessed informed consent before study start. An independent data monitoring committee oversees participant safety and reviews unblinded data.

Vaccine and placebo composition

Each 0.5-ml dose of reconstituted respiratory syncytial virus (RSV) prefusion F protein-based vaccine (RSVPreF3 OA) contained 120 μg of RSVPreF3 antigen and AS01_E_, an adjuvant system containing 25 μg of 3-O-desacyl-4′-monophosphoryl lipid A, 25 μg of QS21 (*Quillaja saponaria* Molina, fraction 21, licensed by GSK from Antigenics LLC, a wholly owned subsidiary of Agenus Inc., a Delaware, USA corporation), and liposome. Placebo was NaCl solution. Both vaccine and placebo were administered by intramuscular injection in the deltoid muscle, preferably of the non-dominant arm.

Vaccine and placebo were prepared and administered by personnel not involved in collecting or evaluating data.

Randomization

Participants were randomly allocated to the trial intervention at dose 1 and dose 2 by means of an automated internet-based system. For the allocation of participants to the first dose of vaccine or placebo (before RSV season 1), the randomization algorithm used a stratification by subset (participants included in the reactogenicity-immunogenicity cohort or not) and a minimization procedure accounting for center, age, and region within each subset.

For the allocation of participants to the RSV_revaccination or RSV_1dose groups (before RSV season 2), the re-randomization algorithm accounted for the stratification and minimization factors used for the initial randomization.

Acute respiratory illness (ARI) surveillance

In this trial, Northern-Hemisphere participants are monitored for the occurrence of ARI for 3 consecutive RSV seasons, Southern-Hemisphere participants for at least 2 seasons.

Participants were instructed to contact the site staff if they experienced at least 2 ARI symptoms/signs lasting ≥24 hours, starting from day 1 (dose 1 administration). Additionally, from day 31, the site staff contacted the participants every 2 weeks during RSV seasons and monthly between seasons to check if the participants had experienced ARI symptoms/signs. When a participant experienced at least 2 ARI symptoms/signs lasting ≥24 hours, an ARI assessment visit was to occur (ideally within 48 hours, maximum 6 days after ARI onset), during which the investigator/staff member evaluated whether the event met the ARI case definition. If it did, a staff member took nasal/throat swabs. Participants also took nasal self-swabs (preferably within 48 hours, maximum 5 days after ARI onset). Further details on surveillance procedures can be found in Papi et al. [1].

Case definitions

***Acute respiratory illness***

An ARI was defined as the presence of:

- At least 2 respiratory symptoms/signs for at least 24 hours

OR

- At least 1 respiratory symptom/sign + 1 systemic symptom/sign for at least 24 hours

Respiratory symptoms/signs were:

- Nasal congestion or rhinorrhea
- Sore throat
- New or increased sputum
- New or increased cough
- New or increased dyspnea (shortness of breath)
- New or increased wheezing (reported by the participant or investigator)
- New or increased crackles/rhonchi (reported by the investigator) based on chest auscultation
- Respiratory rate ≥20 respirations/min (reported by the investigator)
- Low or decreased oxygen saturation (i.e., oxygen saturation <95% or ≤90% if pre-season baseline was <95%, reported by the investigator)
- Need for oxygen supplementation (reported by the investigator)

Systemic symptoms/signs were:

- Fever (body temperature ≥38.0°C/100.4°F by any route) or feverishness (feeling of having fever without objective measurement)
- Fatigue
- Body aches
- Headache
- Decreased appetite

A quantitative reverse transcriptase-polymerase chain reaction (qRT-PCR)-confirmed RSV-ARI was defined as an event meeting the case definition of ARI with at least 1 swab (nasal self-swab or throat or nasal swab taken by trial personnel) positive for RSV-A or RSV-B by qRT-PCR.

***Lower respiratory tract disease (LRTD)***

An LRTD was defined as the presence of:

- At least 2 lower respiratory symptoms/signs for at least 24 hours including at least 1 lower respiratory sign

OR

- At least 3 lower respiratory symptoms for at least 24 hours

Lower respiratory symptoms were:

- New or increased sputum
- New or increased cough
- New or increased dyspnea (shortness of breath)

Lower respiratory signs were:

- New or increased wheezing (reported by the participant or investigator)
- New or increased crackles/rhonchi (reported by the investigator) based on chest auscultation
- Respiratory rate ≥20 respirations/min (reported by the investigator)
- Low or decreased oxygen saturation (i.e., oxygen saturation <95% or ≤90% if pre-season baseline was <95%, reported by the investigator)
- Need for oxygen supplementation (reported by the investigator)

A qRT-PCR-confirmed RSV-LRTD was defined as an event meeting the case definition of LRTD with at least 1 swab (nasal self-swab or throat or nasal swab taken by trial personnel) positive for RSV-A or RSV-B by qRT-PCR.

***Severe RSV-LRTD***

Two different case definitions for severe qRT-PCR-confirmed RSV-LRTD were used:

Definition 1, based on clinical signs or investigator assessment

- An LRTD with at least 1 of the following 2 criteria:
  - At least 2 lower respiratory signs (as listed above)
  - Assessed as ‘severe’ by the investigator

AND

- - With at least 1 swab (nasal self-swab or throat or nasal swab taken by trial personnel) positive for RSV-A or RSV-B by qRT-PCR

Definition 2, based on the receipt supportive therapy*

- An LRTD with at least 1 of the following 3 criteria:
  - Need for oxygen supplementation (reported by the investigator)
  - Need for positive airway pressure therapy (e.g., continuous positive airway pressure)
  - Need for other types of mechanical ventilation

AND

- - With at least 1 swab (nasal self-swab or throat or nasal swab taken by trial personnel) positive for RSV-A or RSV-B by qRT-PCR
- *If the participant was already receiving any of these supportive therapies to treat/control a pre-existing condition, a significant change or adaptation in the therapy was considered as criterion for severe LRTD.

Statistical analyses

Statistical analyses were done by independent external statisticians (to maintain participant-level blinding of the study team) using SAS Life Science Analytics Framework version 5.2.

***Sample size determination and power calculation***

The sample size (target enrollment of up to 25,000 participants) was calculated based on the number of cases needed to demonstrate efficacy of RSVPreF3 OA against RSV-LRTD during the first season, with the criterion of a lower limit of the confidence interval (CI) >20% and an overall significance level of 2.5%.

The power to demonstrate the secondary confirmatory objectives is shown below for the following assumptions:

- a sample size of up to 25,000 participants (23,000 in the Northern and 2000 in the Southern Hemisphere)
- 10% of participants not being evaluable in the efficacy analyses
- a drop-out rate of 20% between each season
- an RSV-LRTD attack rate of 0.6% for each season
- an efficacy against RSV-LRTD of 70% over 1 season after each dose
- an efficacy against RSV-LRTDs of 70% at season 1 and 65% at season 2 after a single dose given before season 1

| **Analysis** | **Expected number of RSV-LRTD cases** | **Power** |
| --- | --- | --- |
| Efficacy of a single dose over 2 seasons, analyzed at the end of Northern-Hemisphere season 2 | 146 | 99.5% |
| Efficacy of annual revaccination over 2 seasons, analyzed at the end of Northern-Hemisphere season 2 | 144 | 99.8% |

Power calculations for a 1-sided alpha of 1.25%, a sample size of 25,000 (23,000 in the Northern and 2000 in the Southern Hemisphere), and an RSV-LRTD attack rate of 0.6% for each season.

RSV-LRTD, respiratory syncytial virus-associated lower respiratory tract disease, confirmed by quantitative reverse transcriptase-polymerase chain reaction.

***Efficacy analyses***

Vaccine efficacy against RSV-LRTD was analyzed overall, by RSV subtype, by participant age category at dose 1, and in participants with/without selected co-existing conditions of interest known to be associated with an increased risk of severe RSV disease. These were cardiorespiratory conditions (chronic respiratory or pulmonary disease [including chronic obstructive pulmonary disease and asthma], chronic heart failure) and endocrine or metabolic conditions (diabetes mellitus type 1 or type 2, advanced liver or renal disease). Vaccine efficacy was also analyzed by frailty status (frail, pre-frail, fit) determined using a gait speed test, in which frail status corresponded to a walking speed of <0.4 m/s or an inability to perform the test, pre-frail corresponded to a walking speed of 0.4–0.99 m/s, and fit status to a walking speed of ≥1 m/s.

For the confirmatory objectives, several sensitivity analyses were performed, including 1 on RSV-LRTD cases without respiratory viral co-infections.

The vaccine efficacy analysis over 2 seasons included season 1 data for participants who received dose 1 (vaccine or placebo) and season 2 data for participants who received dose 1 (vaccine or placebo) and dose 2 (vaccine or placebo). Season 1 data was collected from day 15 post-dose 1 until dose 2 administration or until 30 September 2022 (end of Southern-Hemisphere season 1) for participants who did not receive dose 2. Season 2 data was collected from dose 2 until 31 March 2023 (end of Northern-Hemisphere season 2). For the analysis of a single RSVPreF3 OA dose, participants who received 2 vaccine doses (RSV_revaccination group) contributed to season 1 but were censored before dose 2 administration. For the analysis of the revaccination regimen, participants who received vaccine as dose 1 and placebo as dose 2 (RSV_1dose group) contributed to season 1 but were censored before dose 2 administration.

The vaccine efficacy analysis for season 2 only included data collected from day 15 post-dose 2 until 31 March 2023 (end of Northern-Hemisphere season 2). Participants who had an RSV-ARI case before the start of season 2 (including cases between day 15 post-dose 2 and the start of season 2) were excluded.

The vaccine efficacy analysis of a single RSVPreF3 OA dose over 1 year was based on cases occurring from day 15 post-dose 1 up to 30 September 2022 or dose 2 administration (pre-specified analysis). The analysis until mid-season 2 was performed in a similar way as the analysis over 2 seasons but only included cases accrued until 30 November 2022 (post-hoc analysis).

Periods-at-risk ended at the first occurrence of the event, the data lock point, or drop-out.

Missing or non-evaluable measurements were not imputed. The missing endpoint and censoring are supposed to occur independently, and the pattern of the missingness was either Missing Completely At Random or Missing At Random only.

***Safety analyses***

The numbers and percentages of participants (with exact 95% CIs) with solicited adverse events (AEs), unsolicited AEs, serious AEs (SAEs), and potential immune-mediated diseases (pIMDs) were tabulated for each group. The same was done for solicited and unsolicited AEs with a grade 3 intensity, AEs with a medically attended visit, and AEs considered related to vaccine or placebo administration by the investigator.

For the analysis of solicited AEs, missing or non-evaluable measurements were not replaced; these analyses therefore only included participants with documented solicited safety data available (i.e., paper diary cards completed). For the analysis of unsolicited AEs, SAEs, and pIMDs, all participants who received vaccine or placebo were considered; participants who did not report an event were considered as participants without the event.

Supplementary results

Viral co-infections

During RSV season 1, up to the end of Northern-Hemisphere season 1, 12/47 (25.5%) RSV-LRTD cases had at least 1 viral co-infection (RSVPreF3 OA: 1/7, placebo: 11/40). Up to the end of Southern-Hemisphere season 1, 14/57 (24.6%) RSV-LRTD cases had at least 1 viral co-infection (RSVPreF3 OA: 2/10, placebo: 12/47).

During RSV season 2, 45/131 (34.4%) RSV-LRTD cases had at least 1 viral co-infection (RSV_revaccination: 8/20, RSV_1dose: 7/20; placebo: 30/91).

Safety

Related SAEs and pIMDs were 1 case of anti-neutrophil cytoplasmic antibody positive vasculitis (reported as SAE and pIMD, time-to-onset [TTO]: 64 days); 1 case each of cardiomegaly, pulmonary arterial hypertension, and pulmonary embolism (each reported as SAE in the same participant, TTO: 106 days); 2 cases of cerebrovascular accident (reported as SAE in 2 participants, TTO: 16 and 136 days); 1 case of infection (reported as SAE, TTO: 2 days); 1 case of myocardial infarction (reported as SAE, TTO: 21 days); 1 case of pemphigoid (reported as SAE and pIMD, TTO: 7 days); 1 case of seizure (reported as SAE and pIMD, TTO: 7 days); 1 case of syncope (reported as SAE, TTO: 191 days); and 1 case of psoriasis (reported as pIMD, TTO: 25 days). Group allocation was still blinded for each of these cases.

A numerical imbalance was previously noted in atrial fibrillation occurring within 30 days post-dose 1 (11 [0.09%] RSVPreF3 OA recipients versus 4 [0.03%] placebo recipients, with 8 [0.06%] RSVPreF3 OA recipients versus 1 [0.01%] placebo recipient reporting atrial fibrillation as SAE). The rates of atrial fibrillation reported as SAE within 6 months post-dose 1 were similar in the RSVPreF3 OA group (15 [0.12%] participants) and the placebo group (16 [0.13%] participants). No imbalance in atrial fibrillation was observed within 30 days post-dose 2 (3 [0.06%] participants in RSV_revaccination versus 5 [0.05%] in placebo, with 1 [0.02%] participant in RSV_revaccination and 2 [0.02%] in placebo reporting atrial fibrillation as SAE). A numerical imbalance was observed in atrial fibrillation reported as SAE within 6 months post-dose 2 (11 [0.22%] participants in RSV_revaccination versus 13 [0.13%] in placebo). The Sponsor carried out an in-depth review of atrial fibrillation cases in the trial and concluded that the available data do not support a plausible vaccine-related increased risk.

Supplementary tables

Table S1. Baseline characteristics of the participants (dose 2-exposed population)

| **Characteristic** | **RSVPreF3 OA**  **N=9957** | **RSV_revaccination**  **N=4966** | **RSV_1dose**  **N=4991** | **Placebo**  **N=10,033** |
| --- | --- | --- | --- | --- |
| Mean age (SD), years | 69.4 (6.4) | 69.4 (6.4) | 69.3 (6.4) | 69.5 (6.4) |
| Age group, n (%) |  |  |  |  |
| ≥70 years | 4314 (43.3) | 2167 (43.6) | 2147 (43.0) | 4422 (44.1) |
| ≥80 years | 752 (7.6) | 379 (7.6) | 373 (7.5) | 791 (7.9) |
| 60–69 years | 5643 (56.7) | 2799 (56.4) | 2844 (57.0) | 5611 (55.9) |
| 70–79 years | 3562 (35.8) | 1788 (36.0) | 1774 (35.5) | 3631 (36.2) |
| Sex, n (%) |  |  |  |  |
| Female | 5213 (52.4) | 2570 (51.8) | 2643 (53.0) | 5231 (52.1) |
| Male | 4744 (47.6) | 2396 (48.2) | 2348 (47.0) | 4802 (47.9) |
| Race, n (%) |  |  |  |  |
| Black | 804 (8.1) | 395 (8.0) | 409 (8.2) | 840 (8.4) |
| Asian | 757 (7.6) | 391 (7.9) | 366 (7.3) | 759 (7.6) |
| White | 7936 (79.7) | 3950 (79.5) | 3986 (79.9) | 8009 (79.8) |
| Other | 460 (4.6) | 230 (4.6) | 230 (4.6) | 425 (4.2) |
| Hemisphere^a^, n (%) |  |  |  |  |
| Northern | 9153 (91.9) | 4571 (92.0) | 4582 (91.8) | 9222 (91.9) |
| Southern | 804 (8.1) | 395 (8.0) | 409 (8.2) | 811 (8.1) |
| Type of residence, n (%) |  |  |  |  |
| Community | 9812 (98.5) | 4899 (98.7) | 4913 (98.4) | 9900 (98.7) |
| Long-term care facility | 145 (1.5) | 67 (1.3) | 78 (1.6) | 133 (1.3) |
| Mean BMI (SD), kg/m^2^ | 29.1 (6.0) | 29.0 (6.1) | 29.1 (6.0) | 29.1 (6.0) |
| Frailty status^b^, n (%) |  |  |  |  |
| Frail | 149 (1.5) | 68 (1.4) | 81 (1.6) | 133 (1.3) |
| Pre-frail | 3721 (37.4) | 1884 (37.9) | 1837 (36.8) | 3776 (37.6) |
| Fit | 6074 (61.0) | 3006 (60.5) | 3068 (61.5) | 6109 (60.9) |
| Unknown | 13 (0.1) | 8 (0.2) | 5 (0.1) | 15 (0.1) |
| Co-existing conditions of interest^c^, n (%) |  |  |  |  |
| ≥1condition of interest | 3928 (39.4) | 1947 (39.2) | 1981 (39.7) | 3895 (38.8) |
| Cardiorespiratory condition of interest | 1961 (19.7) | 965 (19.4) | 996 (20.0) | 1953 (19.5) |
| Endocrine or metabolic condition of interest | 2576 (25.9) | 1280 (25.8) | 1296 (26.0) | 2588 (25.8) |

RSVPreF3 OA, group of participants who received a dose of respiratory syncytial virus (RSV) prefusion F protein-based vaccine (RSVPreF3 OA) pre-season 1; RSV_revaccination, group of participants who received a first dose of RSVPreF3 OA pre-season 1 and a second RSVPreF3 OA dose (revaccination) pre-season 2; RSV_1dose, group of participants who received a single RSVPreF3 OA dose pre-season 1 and a placebo dose pre-season 2; placebo, group of participants who received placebo pre-season 1 and pre-season 2; N, number of participants in the dose 2-exposed population; SD, standard deviation; n (%), number (percentage) of participants in the indicated category; BMI, body mass index.

^a^Northern-Hemisphere countries included in the trial are: Belgium, Canada, Estonia, Finland, Germany, Italy, Japan, Mexico, Poland, Republic of Korea, Russian Federation, Spain, United Kingdom, and United States. Southern-Hemisphere countries are: Australia, New Zealand, and South Africa.

^b^Frailty status was assessed using a gait speed test: frail, participants with a walking speed <0.4 m/s or not able to perform the test; pre-frail, participants with a walking speed of 0.4–0.99 m/s; fit, participants with a walking speed ≥1 m/s.

^c^Conditions of interest included any chronic respiratory/pulmonary disease (including chronic obstructive pulmonary disease and asthma) and chronic heart failure (cardiorespiratory), and diabetes mellitus type 1 or type 2 and advanced liver or renal disease (endocrine or metabolic).

Table S2. Vaccine efficacy of a single dose of RSVPreF3 OA against first occurrence of medically attended RSV-LRTD and RSV-ARI over 2 RSV seasons (modified exposed population)

|  | **RSVPreF3 OA (1 dose)** | | | | **Placebo** | | | |  | |
| --- | --- | --- | --- | --- | --- | --- | --- | --- | --- | --- |
| **Endpoint** | **N** | **n** | **T, p-yr** | **n/T,**  **n/1,000 p-yr** | **N** | **n** | **T, p-yr** | **n/T,**  **n/1,000 p-yr** | **Vaccine efficacy,**  **% (95% CI)** | |
| Medically attended RSV-LRTD | 12,469 | 12 | 14,672.1 | 0.8 | 12,498 | 63 | 17,307.8 | 3.6 | 73.1 (49.4–86.9) |  |
| Medically attended RSV-ARI | 12,469 | 32 | 14,661.2 | 2.2 | 12,498 | 94 | 17,287.2 | 5.4 | 52.0 (27.3–69.1) |  |

This post-hoc analysis includes RSV season 1 data from participants who received dose 1 (vaccine or placebo) and season 2 data from participants who received dose 1 (vaccine or placebo) and dose 2 (placebo). Season 1 data was collected from day 15 post-dose 1 until dose 2 administration or until 30 September 2022 (end of Southern-Hemisphere season 1) for participants who did not receive dose 2. Season 2 data was collected from dose 2 until 31 March 2023 (end of Northern-Hemisphere season 2). Participants who received vaccine as dose 1 and placebo as dose 2 (RSV_1dose group) contributed to both seasons. Participants who received 2 vaccine doses (RSV_revaccination group) contributed to season 1 but were censored before dose 2 administration. Vaccine efficacy was estimated using a Poisson model adjusted for age, region, and season.

RSVPreF3 OA, respiratory syncytial virus (RSV) prefusion F protein-based vaccine; RSV-LRTD, RSV-related lower respiratory tract disease confirmed by the adjudication committee; RSV-ARI, RSV-related acute respiratory illness; N, number of participants in the modified exposed population; n, number of participants with ≥1 RSV-LRTD or RSV-ARI with medically attended visit; T, sum of follow-up time (from day 15 post-dose 1 until first occurrence of the event, data lock point, or drop-out); p-yr, person-years; n/T, incidence rate of participants reporting at least 1 event; CI, confidence interval.

Table S3. Vaccine efficacy of a single dose of RSVPreF3 OA and of a first RSVPreF3 OA dose followed by revaccination 1 year later, against first occurrence of RSV-LRTD and RSV-ARI during RSV season 2 only (dose 2-modified exposed population)

|  | **RSVPreF3 OA** | | | | **Placebo** | | | |  |
| --- | --- | --- | --- | --- | --- | --- | --- | --- | --- |
| **Endpoint** | **N** | **n** | **T, p-yr** | **n/T,**  **n/1,000 p-yr** | **N** | **n** | **T, p-yr** | **n/T,**  **n/1,000 p-yr** | **Vaccine efficacy,**  **% (95% CI)** |
| **RSVPreF3 OA 1 dose (pre-season 1)** | | | | | | | | | |
| **RSV-LRTD** |  |  |  |  |  |  |  |  |  |
| Overall | 4991 | 20 | 2448.8 | 8.2 | 10,031 | 91 | 4914.5 | 18.5 | 56.1 (28.2–74.4) |
| Severe^a^ | 4991 | 5 | 2452.6 | 2.0 | 10,031 | 28 | 4933.0 | 5.7 | 64.2 (6.2–89.2) |
| By subtype |  |  |  |  |  |  |  |  |  |
| RSV-A | 4991 | 4 | 2453.1 | 1.6 | 10,031 | 34 | 4930.5 | 6.9 | 76.4 (33.8–93.9) |
| RSV-B | 4991 | 16 | 2449.6 | 6.5 | 10,031 | 57 | 4925.3 | 11.6 | 43.9 (1.0–69.9) |
| By age |  |  |  |  |  |  |  |  |  |
| ≥70 years | 2147 | 8 | 1056.1 | 7.6 | 4420 | 43 | 2174.9 | 19.8 | 62.1 (18.4–84.6) |
| ≥80 years | 373 | 2 | 186.4 | 10.7 | 791 | 7 | 394.1 | 17.8 | 41.0 (-209.9–94.0) |
| 60–69 years | 2844 | 12 | 1392.7 | 8.6 | 5611 | 48 | 2739.6 | 17.5 | 50.9 (6.1–76.3) |
| 70–79 years | 1774 | 6 | 869.7 | 6.9 | 3629 | 36 | 1780.8 | 20.2 | 66.2 (18.9–88.3) |
| By co-existing condition of interest^b^ |  |  |  |  |  |  |  |  |  |
| No condition | 3010 | 8 | 1460.8 | 5.5 | 6136 | 43 | 2973.0 | 14.5 | 62.4 (19.0–84.7) |
| ≥1 condition | 1981 | 12 | 987.9 | 12.1 | 3895 | 48 | 1941.5 | 24.7 | 51.5 (7.4–76.6) |
| ≥1 cardiorespiratory condition | 996 | 7 | 497.0 | 14.1 | 1953 | 39 | 970.8 | 40.2 | 66.5 (24.2–87.4) |
| ≥1 endocrine or metabolic condition | 1296 | 7 | 650.7 | 10.8 | 2588 | 18 | 1298.0 | 13.9 | 23.3 (-92.5–72.9) |
| By frailty^c^ |  |  |  |  |  |  |  |  |  |
| Frail | 81 | *1* | 133 | *1* |  |  |  |  |  |
| Pre-frail | 1837 | 7 | 884.7 | 7.9 | 3775 | 31 | 1815.5 | 17.1 | 53.5 (-7.7–82.7) |
| Fit | 3068 | 12 | 1524.9 | 7.9 | 6108 | 58 | 3026.4 | 19.2 | 59.2 (23.1–80.0) |
| **RSV-ARI** |  |  |  |  |  |  |  |  |  |
| Overall | 4991 | 54 | 2438.1 | 22.1 | 10,031 | 181 | 4888.0 | 37.0 | 40.6 (19.0–57.0) |
| **RSVPreF3 OA dose 1 (pre-season 1) + revaccination (pre-season 2)** | | | | | | | | | |
| **RSV-LRTD** |  |  |  |  |  |  |  |  |  |
| Overall | 4966 | 20 | 2448.2 | 8.2 | 10,031 | 91 | 4914.5 | 18.5 | 55.9 (27.9–74.3) |
| Severe^a^ | 4966 | 5 | 2452.7 | 2.0 | 10,031 | 28 | 4933.0 | 5.7 | 64.1 (5.9–89.2) |
| By subtype |  |  |  |  |  |  |  |  |  |
| RSV-A | 4966 | 11 | 2450.5 | 4.5 | 10,031 | 34 | 4930.5 | 6.9 | 34.9 (-31.6–70.3) |
| RSV-B | 4966 | 9 | 2451.7 | 3.7 | 10,031 | 57 | 4925.3 | 11.6 | 68.4 (35.6–86.2) |
| By age |  |  |  |  |  |  |  |  |  |
| ≥70 years | 2167 | 11 | 1072.0 | 10.3 | 4420 | 43 | 2174.9 | 19.8 | 48.5 (-1.6–76.0) |
| ≥80 years | 379 | 2 | 190.1 | 10.5 | 791 | 7 | 394.1 | 17.8 | 41.9 (-205.2–94.1) |
| 60–69 years | 2799 | 9 | 1376.2 | 6.5 | 5611 | 48 | 2739.6 | 17.5 | 62.5 (22.8–83.8) |
| 70–79 years | 1788 | 9 | 881.9 | 10.2 | 3629 | 36 | 1780.8 | 20.2 | 49.8 (-6.2–78.7) |
| By co-existing condition of interest^b^ |  |  |  |  |  |  |  |  |  |
| No condition | 3019 | 12 | 1469.0 | 8.2 | 6136 | 43 | 2973.0 | 14.5 | 43.3 (-9.4–72.8) |
| ≥1 condition | 1947 | 8 | 979.2 | 8.2 | 3895 | 48 | 1941.5 | 24.7 | 67.1 (29.8–86.6) |
| ≥1 cardiorespiratory condition | 965 | 4 | 489.1 | 8.2 | 1953 | 39 | 970.8 | 40.2 | 79.6 (43.6–94.7) |
| ≥1 endocrine or metabolic condition | 1280 | 6 | 642.8 | 9.3 | 2588 | 18 | 1298.0 | 13.9 | 32.1 (-78.7–77.9) |
| By frailty^c^ |  |  |  |  |  |  |  |  |  |
| Frail | 68 | 0 | 34.0 | 0.0 | 133 | 0 | 65.1 | 0.0 | NE |
| Pre-frail | 1884 | 6 | 907.3 | 6.6 | 3775 | 31 | 1815.5 | 17.1 | 61.7 (6.8–86.9) |
| Fit | 3006 | 14 | 1503.1 | 9.3 | 6108 | 58 | 3026.4 | 19.2 | 51.4 (11.8–75.0) |
| **RSV-ARI** |  |  |  |  |  |  |  |  |  |
| Overall | 4966 | 40 | 2442.4 | 16.4 | 10,031 | 181 | 4888.0 | 37.0 | 55.8 (37.5–69.5) |

Analysis includes data collected from day 15 post-dose 2 (after the start of Northern-Hemisphere RSV season 2) until 31 March 2023 (end of Northern-Hemisphere season 2) from participants who received RSVPreF3 OA as dose 1 and placebo as dose 2 for the analysis of a single dose, from participants who received RSVPreF3 OA as dose 1 and dose 2 for the analysis of the revaccination regimen, and from participants who received placebo as dose 1 and 2 (comparator group for both analyses). Vaccine efficacy was estimated using a Poisson model adjusted for age and region, except for the analysis by age, which used region only as covariate. Participants who had an RSV-ARI case before the start of season 2 (including cases between day 15 post-dose 2 and the start of season 2) were excluded.

RSVPreF3 OA, respiratory syncytial virus (RSV) prefusion F protein-based vaccine; RSV-LRTD, RSV-related lower respiratory tract disease confirmed by the adjudication committee; RSV-ARI, RSV-related acute respiratory illness; N, number of participants in the dose 2-modified exposed population; n, number of participants with ≥1 RSV-LRTD or RSV-ARI (numbers between asterisks, e.g., *1* indicate that data by group are blinded to avoid participant-level unblinding of the study team; the number between the asterisks shows the total number across the 2 groups); T, sum of follow-up time (from day 15 post-dose 2 until first occurrence of the event, data lock point, or drop-out); p-yr, person-years; n/T, incidence rate of participants reporting at least 1 event; CI, confidence interval; NE, could not be estimated.

^a^Severe disease according to either of the 2 case definitions (definition 1 based on clinical signs/investigator assessment or definition 2 based on supportive therapy, see **Supplementary methods**). All severe cases met case definition 1; 1 case in the RSV_1dose group, none in the RSV_revaccination group, and 1 in placebo were confirmed by the adjudication committee as also meeting case definition 2.

^b^Conditions of interest are as explained in Table S1 (footnote).

^c^Frailty status was assessed using a gait speed test as explained in Table S1 (footnote).

Table S4. Most common^a^ serious adverse events within 6 months post-dose 2, classified by MedDRA primary system organ class and high-level term (dose 2-exposed population)

|  | **RSV_revaccination**  **N=4966** | |  | **RSV_1dose**  **N=4991** | |  | **Placebo**  **N=10,033** | |
| --- | --- | --- | --- | --- | --- | --- | --- | --- |
| **Adverse event by system organ class**  and high-level term | **n** | **% (95% CI)** |  | **n** | **% (95% CI)** |  | **n** | **% (95% CI)** |
| **Any serious adverse event** | 210 | 4.2 (3.7–4.8) |  | 219 | 4.4 (3.8–5.0) |  | 461 | 4.6 (4.2–5.0) |
| **Cardiac disorders** | 38 | 0.8 (0.5–1.0) |  | 42 | 0.8 (0.6–1.1) |  | 83 | 0.8 (0.7–1.0) |
| Ischemic coronary artery disorders | 9 | 0.2 (0.1–0.3) |  | 13 | 0.3 (0.1–0.4) |  | 28 | 0.3 (0.2–0.4) |
| Supraventricular arrhythmias | 12 | 0.2 (0.1–0.4) |  | 10 | 0.2 (0.1–0.4) |  | 14 | 0.1 (0.1–0.2) |
| Heart failures NEC | 8 | 0.2 (0.1–0.3) |  | 6 | 0.1 (0.0–0.3) |  | 15 | 0.1 (0.1–0.2) |
| Coronary artery disorders NEC | 6 | 0.1 (0.0–0.3) |  | 5 | 0.1 (0.0–0.2) |  | 15 | 0.1 (0.1–0.2) |
| Ventricular arrhythmias and cardiac arrest | 2 | 0.0 (0.0–0.1) |  | 4 | 0.1 (0.0–0.2) |  | 3 | 0.0 (0.0–0.1) |
| **Infections and infestations** | 38 | 0.8 (0.5–1.0) |  | 48 | 1.0 (0.7–1.3) |  | 77 | 0.8 (0.6–1.0) |
| Lower respiratory tract and lung infections | 15 | 0.3 (0.2–0.5) |  | 18 | 0.4 (0.2–0.6) |  | 24 | 0.2 (0.2–0.4) |
| Coronavirus infections | 2 | 0.0 (0.0–0.1) |  | 7 | 0.1 (0.1–0.3) |  | 10 | 0.1 (0.0–0.2) |
| Sepsis, bacteremia, viremia, and fungemia NEC | 5 | 0.1 (0.0–0.2) |  | 5 | 0.1 (0.0–0.2) |  | 8 | 0.1 (0.0–0.2) |
| Infections NEC | 3 | 0.1 (0.0–0.2) |  | 3 | 0.1 (0.0–0.2) |  | 11 | 0.1 (0.1–0.2) |
| Abdominal and gastrointestinal infections | 5 | 0.1 (0.0–0.2) |  | 7 | 0.1 (0.1–0.3) |  | 4 | 0.0 (0.0–0.1) |
| Urinary tract infections | 6 | 0.1 (0.0–0.3) |  | 5 | 0.1 (0.0–0.2) |  | 4 | 0.0 (0.0–0.1) |
| Bone and joint infections | 0 | 0.0 (0.0–0.1) |  | 2 | 0.0 (0.0–0.1) |  | 6 | 0.1 (0.0–0.1) |
| **Neoplasms benign, malignant, and unspecified (incl cysts and polyps)** | 27 | 0.5 (0.4–0.8) |  | 23 | 0.5 (0.3–0.7) |  | 57 | 0.6 (0.4–0.7) |
| Prostatic neoplasms malignant | 3 | 0.1 (0.0–0.2) |  | 8 | 0.2 (0.1–0.3) |  | 9 | 0.1 (0.0–0.2) |
| Breast and nipple neoplasms malignant | 3 | 0.1 (0.0–0.2) |  | 1 | 0.0 (0.0–0.1) |  | 8 | 0.1 (0.0–0.2) |
| Respiratory tract and pleural neoplasms malignant cell type unspecified NEC | 1 | 0.0 (0.0–0.1) |  | 3 | 0.1 (0.0–0.2) |  | 4 | 0.0 (0.0–0.1) |
| Colorectal neoplasms malignant | 3 | 0.1 (0.0–0.2) |  | 1 | 0.0 (0.0–0.1) |  | 3 | 0.0 (0.0–0.1) |
| Neoplasms malignant site unspecified NEC | 3 | 0.1 (0.0–0.2) |  | 1 | 0.0 (0.0–0.1) |  | 1 | 0.0 (0.0–0.1) |
| **Nervous system disorders** | 20 | 0.4 (0.2–0.6) |  | 23 | 0.5 (0.3–0.7) |  | 52 | 0.5 (0.4–0.7) |
| Central nervous system hemorrhages and cerebrovascular accidents | 9 | 0.2 (0.1–0.3) |  | 9 | 0.2 (0.1–0.3) |  | 27 | 0.3 (0.2–0.4) |
| Disturbances in consciousness NEC | 2 | 0.0 (0.0–0.1) |  | 5 | 0.1 (0.0–0.2) |  | 6 | 0.1 (0.0–0.1) |
| Transient cerebrovascular events | 2 | 0.0 (0.0–0.1) |  | 0 | 0.0 (0.0–0.1) |  | 6 | 0.1 (0.0–0.1) |
| **Injury, poisoning, and procedural complications** | 20 | 0.4 (0.2–0.6) |  | 22 | 0.4 (0.3–0.7) |  | 50 | 0.5 (0.4–0.7) |
| Limb fractures and dislocations | 10 | 0.2 (0.1–0.4) |  | 11 | 0.2 (0.1–0.4) |  | 19 | 0.2 (0.1–0.3) |
| Spinal fractures and dislocations | 0 | 0.0 (0.0–0.1) |  | 3 | 0.1 (0.0–0.2) |  | 9 | 0.1 (0.0–0.2) |
| Non-site-specific injuries NEC | 2 | 0.0 (0.0–0.1) |  | 2 | 0.0 (0.0–0.1) |  | 7 | 0.1 (0.0–0.1) |
| Cerebral injuries NEC | 3 | 0.1 (0.0–0.2) |  | 1 | 0.0 (0.0–0.1) |  | 1 | 0.0 (0.0–0.1) |
| **Gastrointestinal disorders** | 21 | 0.4 (0.3–0.6) |  | 18 | 0.4 (0.2–0.6) |  | 38 | 0.4 (0.3–0.5) |
| Inguinal hernias | 3 | 0.1 (0.0–0.2) |  | 2 | 0.0 (0.0–0.1) |  | 2 | 0.0 (0.0–0.1) |
| Duodenal and small intestinal stenosis and obstruction | 3 | 0.1 (0.0–0.2) |  | 0 | 0.0 (0.0–0.1) |  | 2 | 0.0 (0.0–0.1) |
| **Respiratory, thoracic, and mediastinal disorders** | 16 | 0.3 (0.2–0.5) |  | 24 | 0.5 (0.3–0.7) |  | 34 | 0.3 (0.2–0.5) |
| Bronchospasm and obstruction | 6 | 0.1 (0.0–0.3) |  | 11 | 0.2 (0.1–0.4) |  | 15 | 0.1 (0.1–0.2) |
| Pulmonary thrombotic and embolic conditions | 3 | 0.1 (0.0–0.2) |  | 4 | 0.1 (0.0–0.2) |  | 6 | 0.1 (0.0–0.1) |
| Respiratory failures (excl neonatal) | 1 | 0.0 (0.0–0.1) |  | 5 | 0.1 (0.0–0.2) |  | 3 | 0.0 (0.0–0.1) |
| **Musculoskeletal and connective tissue disorders** | 14 | 0.3 (0.2–0.5) |  | 14 | 0.3 (0.2–0.5) |  | 32 | 0.3 (0.2–0.4) |
| Osteoarthropathies | 4 | 0.1 (0.0–0.2) |  | 7 | 0.1 (0.1–0.3) |  | 19 | 0.2 (0.1–0.3) |
| Musculoskeletal and connective tissue pain and discomfort | 4 | 0.1 (0.0–0.2) |  | 0 | 0.0 (0.0–0.1) |  | 5 | 0.0 (0.0–0.1) |
| **General disorders and administration-site conditions** | 7 | 0.1 (0.1–0.3) |  | 14 | 0.3 (0.2–0.5) |  | 26 | 0.3 (0.2–0.4) |
| Death and sudden death | 5 | 0.1 (0.0–0.2) |  | 6 | 0.1 (0.0–0.3) |  | 10 | 0.1 (0.0–0.2) |
| Pain and discomfort NEC | 2 | 0.0 (0.0–0.1) |  | 4 | 0.1 (0.0–0.2) |  | 11 | 0.1 (0.1–0.2) |
| **Vascular disorders** | 6 | 0.1 (0.0–0.3) |  | 6 | 0.1 (0.0–0.3) |  | 29 | 0.3 (0.2–0.4) |
| Peripheral vasoconstriction, necrosis, and vascular insufficiency | 1 | 0.0 (0.0–0.1) |  | 2 | 0.0 (0.0–0.1) |  | 10 | 0.1 (0.0–0.2) |
| **Renal and urinary disorders** | 6 | 0.1 (0.0–0.3) |  | 9 | 0.2 (0.1–0.3) |  | 18 | 0.2 (0.1–0.3) |
| Renal failure and impairment | 3 | 0.1 (0.0–0.2) |  | 5 | 0.1 (0.0–0.2) |  | 9 | 0.1 (0.0–0.2) |
| **Metabolism and nutrition disorders** | 5 | 0.1 (0.0–0.2) |  | 5 | 0.1 (0.0–0.2) |  | 16 | 0.2 (0.1–0.3) |
| **Hepatobiliary disorders** | 7 | 0.1 (0.1–0.3) |  | 4 | 0.1 (0.0–0.2) |  | 11 | 0.1 (0.1–0.2) |
| Cholecystitis and cholelithiasis | 4 | 0.1 (0.0–0.2) |  | 4 | 0.1 (0.0–0.2) |  | 8 | 0.1 (0.0–0.2) |
| **Psychiatric disorders** | 3 | 0.1 (0.0–0.2) |  | 4 | 0.1 (0.0–0.2) |  | 13 | 0.1 (0.1–0.2) |
| **Eye disorders** | 9 | 0.2 (0.1–0.3) |  | 5 | 0.1 (0.0–0.2) |  | 4 | 0.0 (0.0–0.1) |
| Cataract conditions | 5 | 0.1 (0.0–0.2) |  | 3 | 0.1 (0.0–0.2) |  | 3 | 0.0 (0.0–0.1) |
| **Reproductive system and breast disorders** | 5 | 0.1 (0.0–0.2) |  | 3 | 0.1 (0.0–0.2) |  | 7 | 0.1 (0.0–0.1) |
| **Blood and lymphatic system disorders** | 3 | 0.1 (0.0–0.2) |  | 3 | 0.1 (0.0–0.2) |  | 4 | 0.0 (0.0–0.1) |
| **Investigations** | 2 | 0.0 (0.0–0.1) |  | 3 | 0.1 (0.0–0.2) |  | 2 | 0.0 (0.0–0.1) |
| **Skin and subcutaneous tissue disorders** | 3 | 0.1 (0.0–0.2) |  | 1 | 0.0 (0.0–0.1) |  | 3 | 0.0 (0.0–0.1) |

RSV_revaccination, group of participants who received a first dose of respiratory syncytial virus (RSV) prefusion F protein-based vaccine (RSVPreF3 OA) pre-season 1 and a second RSVPreF3 OA dose (revaccination) pre-season 2; RSV_1dose, group of participants who received a single RSVPreF3 OA dose pre-season 1 and a placebo dose pre-season 2; placebo, group of participants who received placebo pre-season 1 and pre-season 2; MedDRA, Medical Dictionary for Regulatory Activities; N, number of participants in the dose 2-exposed population; n/%, number/percentage of participants presenting at least 1 type of adverse event; CI, confidence interval; NEC, not elsewhere coded.

^a^Table shows system organ classes and high-level terms that were reported as serious adverse event for ≥0.1% of participants in any of the groups.

Table S5. Most common^a^ potential immune-mediated diseases with onset within 6 months post-dose 2, classified by MedDRA primary system organ class and high-level term (dose 2-exposed population)

|  | **RSV_revaccination**  **N=4966** | |  | **RSV_1dose**  **N=4991** | |  | **Placebo**  **N=10,033** | |
| --- | --- | --- | --- | --- | --- | --- | --- | --- |
| **Adverse event by system organ class**  and high-level term | **n** | **% (95% CI)** |  | **n** | **% (95% CI)** |  | **n** | **% (95% CI)** |
| **Any event** | 14 | 0.3 (0.2–0.5) |  | 19 | 0.4 (0.2–0.6) |  | 35 | 0.3 (0.2–0.5) |
| **Musculoskeletal and connective tissue disorders** | 3 | 0.1 (0.0–0.2) |  | 2 | 0.0 (0.0–0.1) |  | 6 | 0.1 (0.0–0.1) |
| Connective tissue disorders NEC | 1 | 0.0 (0.0–0.1) |  | 1 | 0.0 (0.0–0.1) |  | 3 | 0.0 (0.0–0.1) |
| **Skin and subcutaneous tissue disorders** | 4 | 0.1 (0.0–0.2) |  | 4 | 0.1 (0.0–0.2) |  | 2 | 0.0 (0.0–0.1) |
| Psoriatic conditions | 2 | 0.0 (0.0–0.1) |  | 1 | 0.0 (0.0–0.1) |  | 1 | 0.0 (0.0–0.1) |
| **Metabolism and nutrition disorders** | 3 | 0.1 (0.0–0.2) |  | 0 | 0.0 (0.0–0.1) |  | 6 | 0.1 (0.0–0.1) |
| Disorders of purine metabolism | 3 | 0.1 (0.0–0.2) |  | 0 | 0.0 (0.0–0.1) |  | 5 | 0.0 (0.0–0.1) |
| **Respiratory, thoracic, and mediastinal disorders** | 1 | 0.0 (0.0–0.1) |  | 1 | 0.0 (0.0–0.1) |  | 5 | 0.0 (0.0–0.1) |
| Parenchymal lung disorders NEC | 1 | 0.0 (0.0–0.1) |  | 1 | 0.0 (0.0–0.1) |  | 4 | 0.0 (0.0–0.1) |
| **Gastrointestinal disorders** | 1 | 0.0 (0.0–0.1) |  | 3 | 0.1 (0.0–0.2) |  | 2 | 0.0 (0.0–0.1) |
| Colitis (excl infective) | 1 | 0.0 (0.0–0.1) |  | 2 | 0.0 (0.0–0.1) |  | 2 | 0.0 (0.0–0.1) |
| **Vascular disorders** | 1 | 0.0 (0.0–0.1) |  | 1 | 0.0 (0.0–0.1) |  | 3 | 0.0 (0.0–0.1) |
| **Cardiac disorders** | 0 | 0.0 (0.0–0.1) |  | 3 | 0.1 (0.0–0.2) |  | 1 | 0.0 (0.0–0.1) |

RSV_revaccination, group of participants who received a first dose of respiratory syncytial virus (RSV) prefusion F protein-based vaccine (RSVPreF3 OA) pre-season 1 and a second RSVPreF3 OA dose (revaccination) pre-season 2; RSV_1dose, group of participants who received a single RSVPreF3 OA dose pre-season 1 and a placebo dose pre-season 2; placebo, group of participants who received placebo pre-season 1 and pre-season 2; MedDRA, Medical Dictionary for Regulatory Activities; N, number of participants in the dose 2-exposed population; n/%, number/percentage of participants presenting at least 1 type of adverse event; CI, confidence interval; NEC, not elsewhere coded.

^a^Table shows system organ classes and high-level terms that were reported as potential immune-mediated disease for ≥4 participants in total (across groups).

Table S6. Most common^a^ fatal serious adverse events from dose 2 administration until the end of season 2, classified by MedDRA primary system organ class and high-level term (dose 2-exposed population)

|  | **RSV_revaccination**  **N=4966** | |  | **RSV_1dose**  **N=4991** | |  | **Placebo**  **N=10,033** | |
| --- | --- | --- | --- | --- | --- | --- | --- | --- |
| **Adverse event by system organ class**  and high-level term | **n** | **% (95% CI)** |  | **n** | **% (95% CI)** |  | **n** | **% (95% CI)** |
| **Any fatal serious adverse event** | 20 | 0.4 (0.2–0.6) |  | 26 | 0.5 (0.3–0.8) |  | 41 | 0.4 (0.3–0.6) |
| **General disorders and administration-site conditions** | 5 | 0.1 (0.0–0.2) |  | 6 | 0.1 (0.0–0.3) |  | 13 | 0.1 (0.1–0.2) |
| Death and sudden death | 5 | 0.1 (0.0–0.2) |  | 6 | 0.1 (0.0–0.3) |  | 12 | 0.1 (0.1–0.2) |
| **Cardiac disorders** | 6 | 0.1 (0.0–0.3) |  | 4 | 0.1 (0.0–0.2) |  | 12 | 0.1 (0.1–0.2) |
| Heart failures NEC | 2 | 0.0 (0.0–0.1) |  | 1 | 0.0 (0.0–0.1) |  | 4 | 0.0 (0.0–0.1) |
| Ventricular arrhythmias and cardiac arrest | 1 | 0.0 (0.0–0.1) |  | 3 | 0.1 (0.0–0.2) |  | 3 | 0.0 (0.0–0.1) |
| Ischemic coronary artery disorders | 2 | 0.0 (0.0–0.1) |  | 1 | 0.0 (0.0–0.1) |  | 3 | 0.0 (0.0–0.1) |
| **Infections and infestations** | 2 | 0.0 (0.0–0.1) |  | 7 | 0.1 (0.1–0.3) |  | 4 | 0.0 (0.0–0.1) |
| Lower respiratory tract and lung infections | 1 | 0.0 (0.0–0.1) |  | 3 | 0.1 (0.0–0.2) |  | 1 | 0.0 (0.0–0.1) |
| Sepsis, bacteremia, viremia, and fungemia NEC | 1 | 0.0 (0.0–0.1) |  | 2 | 0.0 (0.0–0.1) |  | 2 | 0.0 (0.0–0.1) |
| **Neoplasms benign, malignant, and unspecified (incl cysts and polyps)** | 2 | 0.0 (0.0–0.1) |  | 4 | 0.1 (0.0–0.2) |  | 6 | 0.1 (0.0–0.1) |
| **Nervous system disorders** | 2 | 0.0 (0.0–0.1) |  | 3 | 0.1 (0.0–0.2) |  | 3 | 0.0 (0.0–0.1) |
| Central nervous system hemorrhages and cerebrovascular accidents | 2 | 0.0 (0.0–0.1) |  | 3 | 0.1 (0.0–0.2) |  | 1 | 0.0 (0.0–0.1) |
| **Respiratory, thoracic, and mediastinal disorders** | 1 | 0.0 (0.0–0.1) |  | 3 | 0.1 (0.0–0.2) |  | 2 | 0.0 (0.0–0.1) |
| **Injury, poisoning, and procedural complications** | 0 | 0.0 (0.0–0.1) |  | 1 | 0.0 (0.0–0.1) |  | 4 | 0.0 (0.0–0.1) |

RSV_revaccination, group of participants who received a first dose of respiratory syncytial virus (RSV) prefusion F protein-based vaccine (RSVPreF3 OA) pre-season 1 and a second RSVPreF3 OA dose (revaccination) pre-season 2; RSV_1dose, group of participants who received a single RSVPreF3 OA dose pre-season 1 and a placebo dose pre-season 2; placebo, group of participants who received placebo pre-season 1 and pre-season 2; MedDRA, Medical Dictionary for Regulatory Activities; N, number of participants in the dose 2-exposed population; n/%, number/percentage of participants presenting at least 1 type of adverse event; CI, confidence interval; NEC, not elsewhere coded.

^a^Table shows system organ classes and high-level terms that were reported as fatal serious adverse event for ≥4 participants in total (across groups).

Supplementary references

1. Papi A, Ison MG, Langley JM, et al. Respiratory Syncytial Virus Prefusion F Protein Vaccine in Older Adults. N Engl J Med **2023**; 388:595-608.
